# Comparative physiological and metabolomic analyses reveal that Fe_3_O_4_ and ZnO nanoparticles alleviate Cd toxicity in tobacco

**DOI:** 10.1186/s12951-022-01509-3

**Published:** 2022-06-27

**Authors:** Congming Zou, Tianquan Lu, Ruting Wang, Peng Xu, Yifen Jing, Ruling Wang, Jin Xu, Jinpeng Wan

**Affiliations:** 1 Yunnan Academy of Tobacco Agricultural Sciences, Kunming, 650021 Yunnan China; 2 CAS Key Laboratory of Tropical Plant Resources and Sustainable Use, Xishuangbanna Tropical Botanical Garden, Chinese Academy of Sciences, Menglun, Mengla, 666303 Yunnan China; 3 College of Horticulture, Shanxi Agricultural University, Taigu, 030801 Shanxi China; 4 Center of Economic Botany, Chinese Academy of Sciences, Menglun, Mengla, 666303 Yunnan China; 5 University of Chinese Academy of Sciences, Beijing, 100049 China

**Keywords:** Metabolomics, Cadmium, Alkaloids, Amino acids, Flavonoids

## Abstract

**Background:**

Heavy metals repress tobacco growth and quality, and engineered nanomaterials have been used for sustainable agriculture. However, the underlying mechanism of nanoparticle-mediated cadmium (Cd) toxicity in tobacco remains elusive.

**Results:**

Herein, we investigated the effects of Fe_3_O_4_ and ZnO nanoparticles (NPs) on Cd stress in tobacco cultivar ‘Yunyan 87’ (*Nicotiana* *tabacum*). Cd severely repressed tobacco growth, whereas foliar spraying with Fe_3_O_4_ and ZnO NPs promoted plant growth, as indicated by enhancing plant height, root length, shoot and root fresh weight under Cd toxicity. Moreover, Fe_3_O_4_ and ZnO NPs increased, including Zn, K and Mn contents, in the roots and/or leaves and facilitated seedling growth under Cd stress. Metabolomics analysis showed that 150 and 76 metabolites were differentially accumulated in roots and leaves under Cd stress, respectively. These metabolites were significantly enriched in the biosynthesis of amino acids, nicotinate and nicotinamide metabolism, arginine and proline metabolism, and flavone and flavonol biosynthesis. Interestingly, Fe_3_O_4_ and ZnO NPs restored 50% and 47% in the roots, while they restored 70% and 63% in the leaves to normal levels, thereby facilitating plant growth. Correlation analysis further indicated that these metabolites, including proline, 6-hydroxynicotinic acid, farrerol and quercetin-3-*O*-sophoroside, were significantly correlated with plant growth.

**Conclusions:**

These results collectively indicate that metal nanoparticles can serve as plant growth regulators and provide insights into using them for improving crops in heavy metal-contaminated areas.

**Graphical Abstract:**

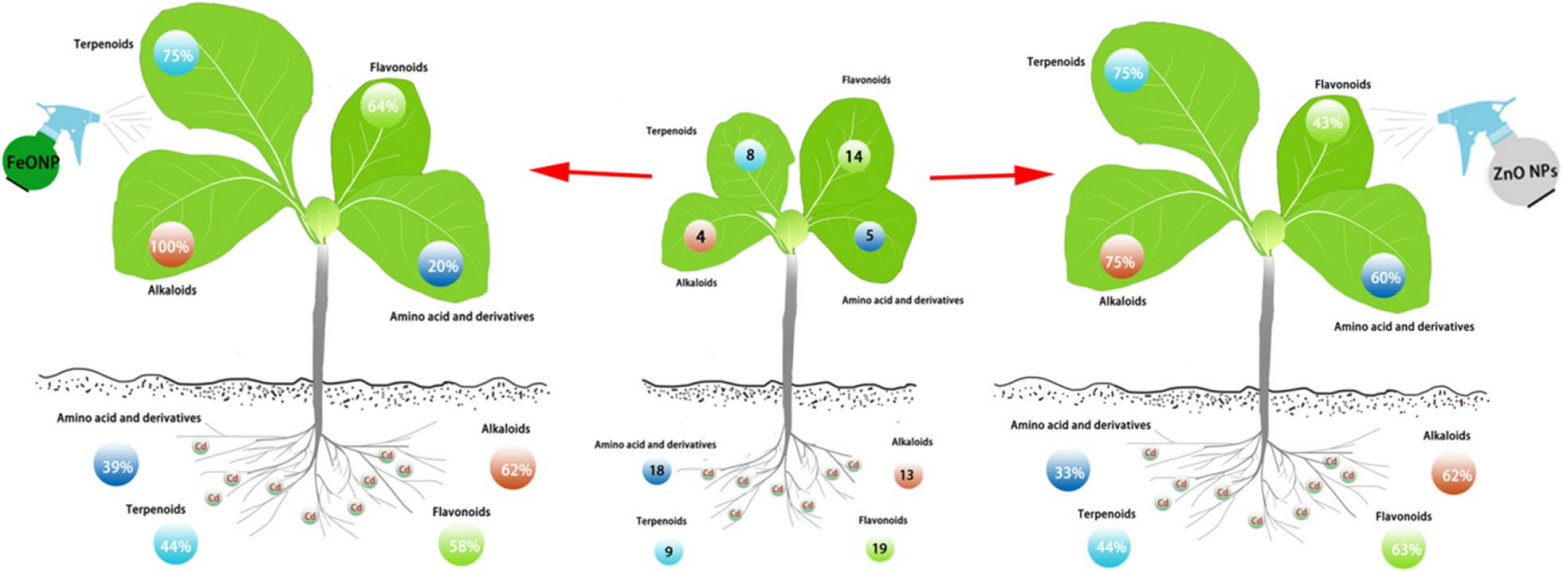

**Supplementary Information:**

The online version contains supplementary material available at 10.1186/s12951-022-01509-3.

## Background

Toxic heavy metals have been attracted public attention for their significant environmental and human health threat [1, 2]. In addition, they severely affect plant growth, quality, and yield [3]. Therefore, plants have adopted efficient strategies incorporating accumulation and non-accumulation mechanisms to detoxify heavy metals over long-term evolution [4, 5]. Phytochelatins and sequestration in vacuoles are among the most prevalent ways to reduce toxic metal translocation [6, 7]. However, anthropogenic activities have increased environmental pollution problems. Therefore, it becomes urgent to cope with toxic heavy metals in crops.

Nanotechnology has been extensively used in industry, agriculture, and the environment [8–11]. However, engineered nanomaterials (EMs) negatively or positively affect plant growth and development attributed to their physiochemical properties, application, and concentrations. In particular, nano-based materials show great potential in crop improvement under heavy metals [12–15].

Iron-based nanomaterials, widely used as adsorbents to remove heavy metals from sewage and sludge, have recently attracted extensive attention [14, 16, 17]. Li et al. [18] observed that γ-Fe_2_O_3_ NPs affected corn seed germination and antioxidant activity. In addition, nano-Fe_2_O_3_ acted as nanofertilizers to promote plant growth and enhance photosynthetic efficiency [19, 20]. On the other hand, Fe_3_O_4_ NPs caused more significant oxidative damage than bulked Fe_3_O_4_ in plants [21]. Iron-based nanomaterials also showed promising applications for protecting plants against abiotic stress. Nano-Fe_3_O_4_ alleviated the effects of Cd on growth and yield parameters [22], and they also decreased Cd-induced growth inhibition by modulating antioxidant capacity [23, 24].

Zinc oxide nanoparticles (ZnO NPs) exert positive, negative, or neutral effects on plants [25, 26]. Controversial results have demonstrated different effects of ZnO NPs and zinc ions (Zn^2+^) on plants. The ZnO NPs toxicity is attributed to NPs themselves or released excess ions from NPs [27, 28]. However, studies have also indicated that ZnO NPs and Zn^2+^ toxicity are significantly different [29, 30]. Although ZnO NPs show a more substantial inhibitory effect on plant growth at a lower dosage than Zn^2+^, ZnO NPs restore more rapid plant growth than Zn^2+^ [30]. Moreover, studies have revealed the roles of ZnO NPs in stress responses in recent decades. ZnO NPs reprogram carbon/nitrogen and secondary metabolism, thus effectively relieving iron deficiency-induced chlorosis and enhancing salt tolerance [31, 32]. Furthermore, ZnO NPs have been used to cope with heavy metals in plants. For example, Rizwan et al. [33] demonstrate that ZnO NPs can alleviate Cd toxicity in maize cultivated in contaminated soil. ZnO NPs also enable the potential to decrease Cd and arsenic accumulation in rice [34, 35]. The mechanism of the ZnO NP-mediated response to heavy metals in tobacco is still worthy of further exploration.

Tobacco (*Nicotiana* *tabacum* L.), a commercially cultivated model plant worldwide, has attracted extensive attention for its molecular regulation of flower coloration [36, 37]. It has also drawn the interest of scientists due to its relative tolerance of Cd [38, 39], and genetic modification has been adopted to reduce Cd uptake by tobacco [40, 41]. Zhang et al. [42] show that Cd uptake, sequestration, remobilization, and chelation ability differ in two contrasting tobacco species. The specific expression of *ZIP* genes mediated the accumulation of Zn and Cd in tobacco [43]. Fortunately, EMs play essential roles in suppressing Turnip mosaic virus infection in tobacco [44]. Alkhatib et al. [45] demonstrate that Fe_3_O_4_ NPs affect tobacco seed germination in size- and dosage-dependent manners. However, little attention has been paid to EMs mediated tobacco response to heavy metals. Therefore, it is imperative to implement effective strategies, including using iron- and zinc-based materials, for the sustainable development of the tobacco industry.

Using the tobacco cultivar ‘Yunyan 87’ (*N.* *tabacum*), we investigated the mechanisms of NP-mediated Cd tolerance in plants. Our results indicated that Cd inhibited the growth of tobacco seedlings. In addition, exogenous applications of Fe_3_O_4_ and ZnO NPs affected nutrient element uptake. Subsequently, metabolomics analyses revealed the mechanism of Fe_3_O_4_ NP- and ZnO NP-mediated Cd toxicity tolerance in tobacco seedlings. This study suggests that metal nanoparticles may serve as plant growth regulators and provide new insight into using metal nanoparticles to improve crops in toxic metal-contaminated areas.

## Results

### Determination of Fe_3_O_4_ and ZnO nanoparticles

The average hydrodynamic size and zeta potential for Fe_3_O_4_ NPs were 637.87 ± 42.35 nm and 2.71 ± 0.08 mV, respectively (Additional file 1: Fig. S1), while the average hydrodynamic size and zeta potential for ZnO NPs were 278.23 ± 12.72 nm and 3.09 ± 0.71 mV, respectively (Additional file 1: Fig. S1). We further analyzed the ions released from the NPs. The Fe or Zn contents of 50 mg·L^−1^ Fe_3_O_4_ NPs or ZnO NPs were 0.23 mg·L^−1^ and 0.87 mg·L^−1^, respectively (Additional file 1: Fig. S2), indicating a relatively low concentration of ions released from the NPs.

### Fe_3_O_4_ and ZnO NPs affect plant growth and mineral element accumulation in tobacco

**Fig. 1:**
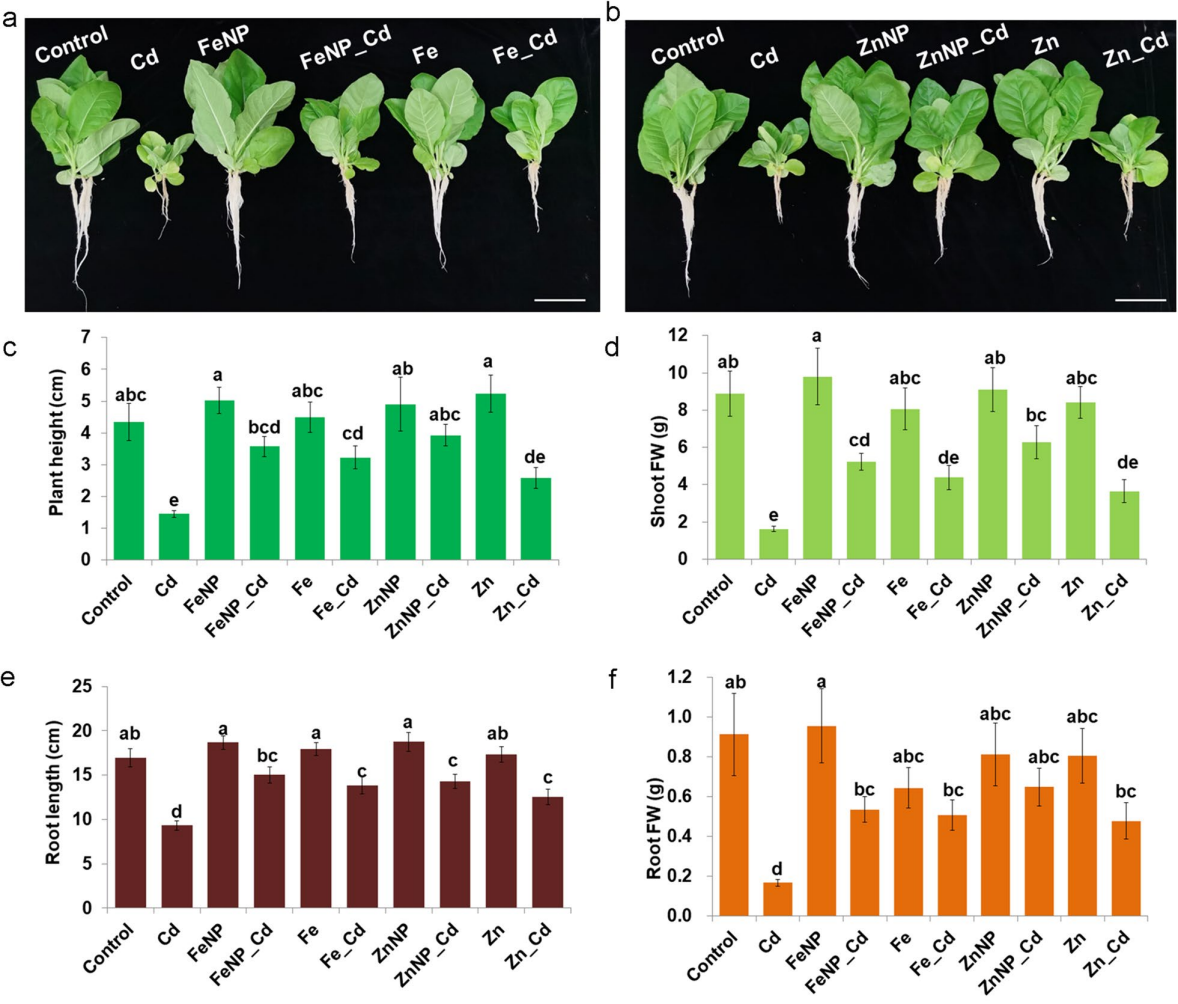
Effects of Fe_3_O_4_ and ZnO NPs on the growth of tobacco. Representative phenotypes of Fe_3_O_4_ NPs (**a**) and ZnO NPs (**b**) supplemented with or without 5 μM Cd. **c**, **d** The plant height and shoot fresh weight of ‘Yunyan 87’ seedlings; **e**, **f** The root length and fresh weight of tobacco seedlings. FW, fresh weight. Bars = 5 cm. The results shown are the means ± SE (*n* = 3; 15 plants/treatment/replicate), and different letters show significantly different values (p < 0.05 according to Tukey’s test)

We first determined the effects of Cd on tobacco seedling growth. We found that Cd treatment markedly induced growth inhibition in plant height, shoot FW, root length and FW (Fig. 1). To confirm whether Fe_3_O_4_ and ZnO NPs played roles in mediating Cd response, we further applied foliar exposure to 50 mg·L^−1^ Fe_3_O_4_, ZnO NPs, and ion solutions to tobacco seedlings. The foliar applications of NPs and ions had no side effects on the plant height, shoot FW, root length and FW under untreated conditions (Fig. 1).

Compared with Cd-treated seedlings alone, Fe_3_O_4_, ZnO NPs, and ions significantly promoted root growth and FW. Both Fe_3_O_4_ and ZnO NPs enhanced plant growth, as indicated by higher plant height and shoot FW; however, FeSO_4_ elevated plant height but had no effects on the shoot FW. Similarly, ZnSO_4_ showed no noticeable promotion effects on the plant height and shoot FW (Fig. 1). These results suggested that moderate concentrations of NPs and ions played different roles in tobacco seedlings under Cd stress, and NPs were more efficient than ions.

**Fig. 2:**
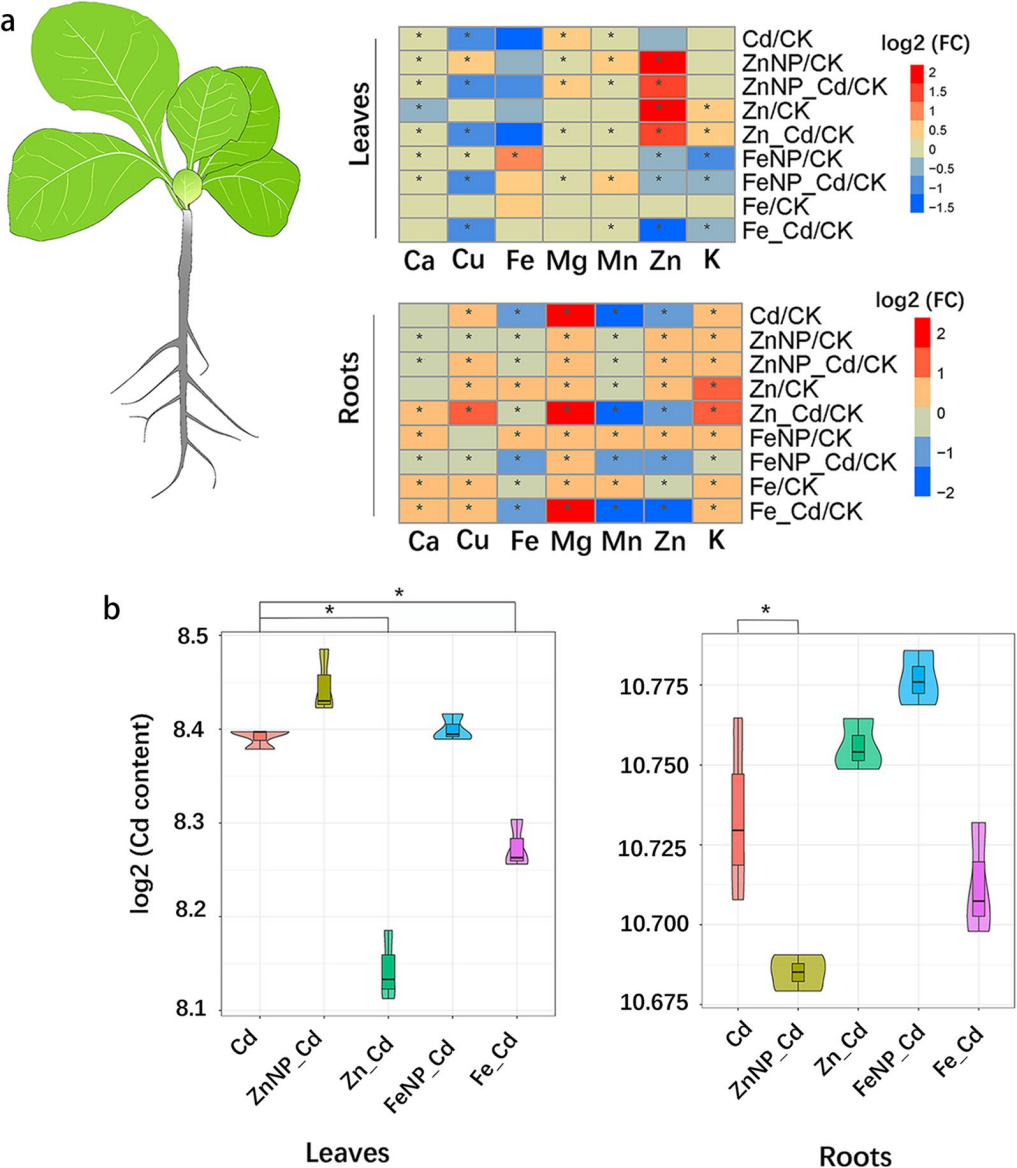
Effects of Fe_3_O_4_ and ZnO NPs on nutrient element accumulation of tobacco. Heat maps showed the log_2_FC value of the different treatments compared with the control (**a**). The violin plot showed log_2_ Cd content under Cd stress conditions (**b**). The results shown are the means ± SE (*n* = 3; 15 plants/treatment/replicate), and the different letters indicate significantly different values (*P* < 0.05 according to Tukey’s test). *Significantly different values at *P* < 0.05 according to a Student’s *t*-test

Cd toxicity severely suppresses plant growth and ion balance, thus further disrupting plant metabolism and energy metabolism. We thus determined the elements in the leaves and roots. Cd treatment markedly reduced the contents of Fe, Mn and Zn but increased the Cu, Mg and K in the roots (Fig. 2a). Cd treatment also reduced the contents of Ca, Cu and Mn but increased Mg in the leaves (Fig. 2a). Under Cd stress, mineral element uptake and distribution were altered. Fe_3_O_4_ NPs or FeSO_4_ increased Mg but reduced Fe, Mn and Zn in the roots and decreased Cu, Zn and K in the leaves. In addition, ZnO NPs or ZnSO_4_ increased Cu, Mg and K but decreased Fe and Mn in the roots; however, they both increased Zn but reduced Ca, Cu and Mn in the leaves (Fig. 2a). In addition, FeSO_4_ or ZnSO_4_ decreased the Cd in the leaves, while ZnO NPs reduced it in the roots (Fig. 2b). These results showed that NPs and ions affected the balance of the element in tobacco seedlings.

### Metabolomics profiling analysis

We examined differentially accumulated metabolites (DAMs) in the roots and leaves for the metabolomic profile. Compared to untreated conditions, metabolites in roots or leaves exposed to Fe_3_O_4_, ZnO NPs, FeSO_4_ ZnSO_4_, Cd toxicity, or their combinations presented a clear separation (Additional file 1: Figs. S3–5). This result indicated that Cd stress reprogrammed the metabolites, and NPs or ions altered the metabolites in the roots and leaves of untreated or Cd-exposed tobacco seedlings.

**Fig. 3:**
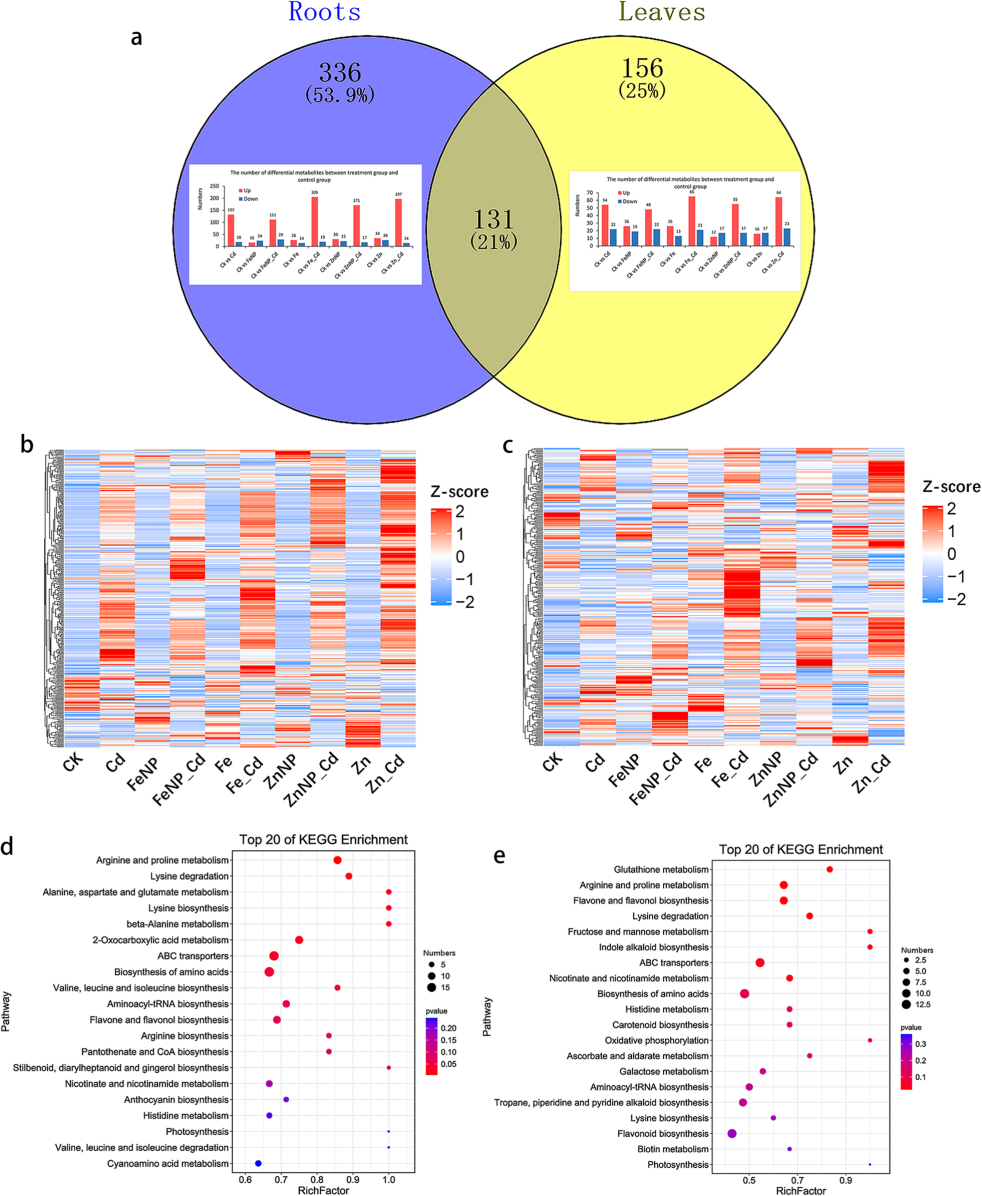
Differentially accumulated metabolites among various treatments. DAMs in roots and leaves under different treatments (**a**). Heat maps of the detected metabolites in the roots (**b**) and leaves (**c**), respectively. Top 20 enriched DAMs in the roots (**d**) and leaves (**e**)

A total of 1013 and 890 metabolites were identified in tobacco roots and leaves, respectively. Then, a VIP ≥ 1 and *P*-value < 0.05 were set as the threshold of DAM characterization. As a result, 467 and 287 DAMs were identified in tobacco roots and leaves, respectively (Fig. 3a, Additional file 2: Table S1 and Additional file 3: Table S2). Among them, 131 DAMs were determined in both roots and leaves (Fig. 3, Additional file 2: Table S1 and Additional file 3: Table S2). Furthermore, several of the top 20 pathways, including arginine and proline metabolism, amino acid biosynthesis, nicotinate and nicotinamide metabolism, were significantly enriched in tobacco roots and leaves (Fig. 3d, e).

Under Cd stress, a total of 150 DAMs, including increases 132 and decreases 18 in tobacco roots, and 76 DAMs, including increases 54 and decreases 22 in tobacco leaves, were identified (Cd/control) (Fig. 3a, Additional file 2: Table S1 and Additional file 3: Table S2).

Fe_3_O_4_ NPs increased 16 DAMs and decreased 24 DAMs in tobacco roots, while increased 26 DAMs and decreased 19 DAMs in tobacco leaves (FeNP/control) (Fig. 3a, Additional file 2: Table S1 and Additional file 3: Table S2). FeSO_4_ increased 26 DAMs and decreased 14 DAMs in tobacco roots, while increased 26 DAMs and decreased 13 DAMs in tobacco leaves (Fe/control) (Fig. 3a, Additional file 2: Table S1 and Additional file 3: Table S2).

ZnO NPs increased 30 DAMs and decreased 22 DAMs in tobacco roots, while increased 12 DAMs and decreased 17 DAMs in tobacco leaves (ZnNP/control) (Fig. 3a, Additional file 2: Table S1 and Additional file 3: Table S2). On the other hand, ZnSO_4_ increased 34 DAMs and decreased 26 DAMs in tobacco roots, while increased 16 DAMs and decreased 17 DAMs in tobacco leaves (Zn/control) (Fig. 3a, Additional file 2: Table S1 and Additional file 3: Table S2).

Compared with Cd stress alone, Fe_3_O_4_ NPs resulted in 37 DAMs, including increased 10 and decreased 27, and 48 DAMs, including increased 30 and decreased 18, in Cd-treated tobacco roots and leaves (FeNP_Cd/Cd), respectively (Additional file 2: Table S1 and Additional file 3: Table S2). While FeSO_4_ resulted in 46 DAMs (37 showed increased levels and 9 showed decreased levels) and 72 DAMs (53 showed increased levels and 19 showed decreased levels) in the Cd-treated tobacco roots and leaves (Fe_Cd/Cd), respectively (Additional file 2: Table S1 and Additional file 3: Table S2).

ZnO NPs resulted in 50 DAMs, including increased 35 and decreased 15, and 47 DAMs, including increased 32 and decreased 15, in the Cd-treated tobacco roots and leaves (ZnNP_Cd/Cd), respectively (Additional file 2: Table S1 and Additional file 3: Table S2). On the other hand, ZnSO_4_ resulted in 68 DAMs, including increased 56 and decreased 12, and 64 DAMs, including increased 45 and decreased 19, in the Cd-treated tobacco roots and leaves (Zn_Cd/Cd), respectively (Additional file 2: Table S1 and Additional file 3: Table S2).

**Fig. 4:**
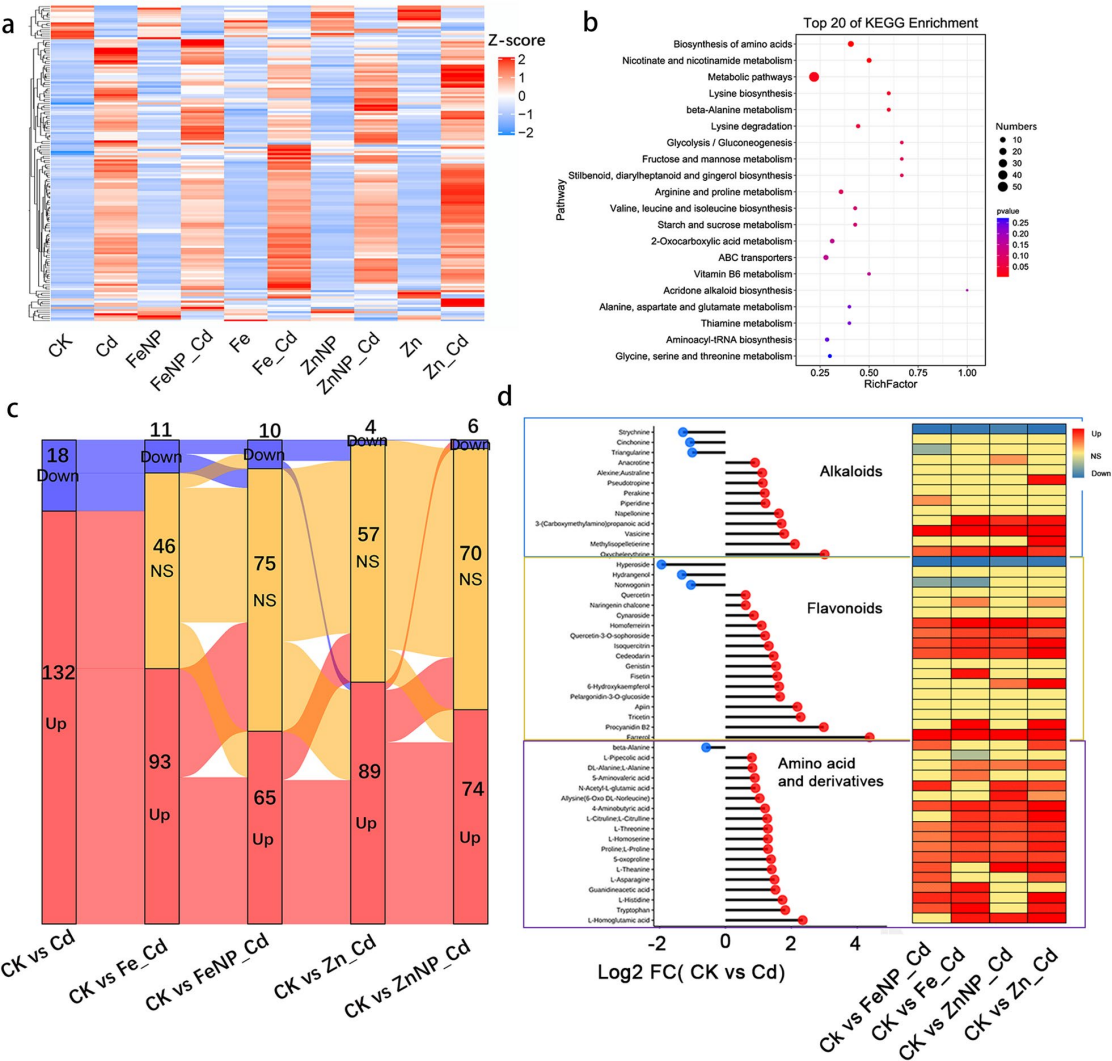
Cd-induced differentially accumulated metabolites in the roots. The heat maps of Cd-induced DAMs content (Z-score value) in the roots among different treatments (**a**). Top 20 KEGG enrichment of DAMs (**b**). Sankey diagram of DAMs among various treatments compared with the control (**c**). The significant differentially accumulated alkaloids, flavonoids, amino acids and derivatives responses to Cd (**d**). *Up* upregulated metabolites; *Down* downregulated metabolites; and *NS* non-significant metabolites

**Fig. 5:**
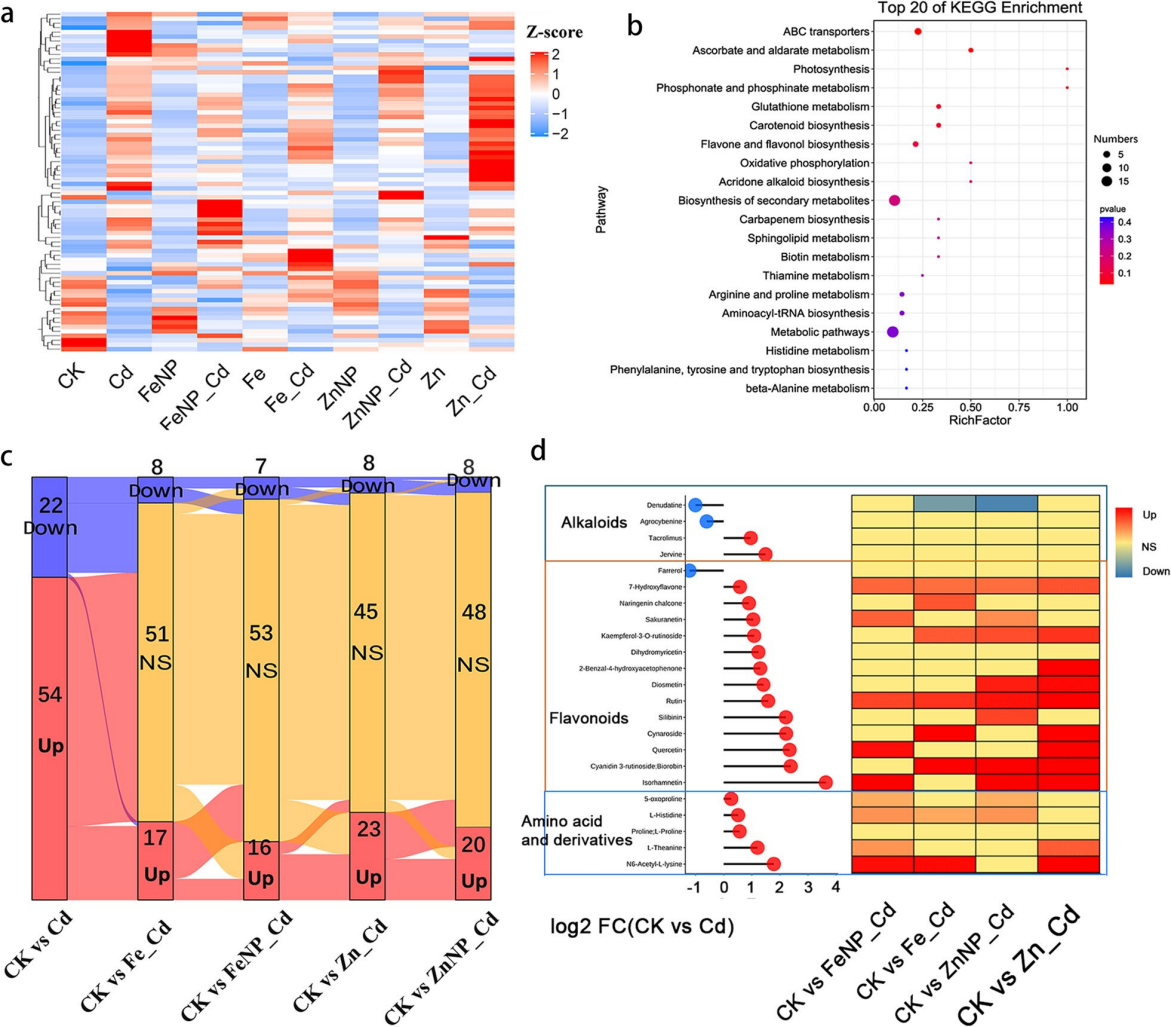
Cd-induced differentially accumulated metabolites in the leaves. The heat maps of Cd-induced DAMs content (Z-score value) in the leaves among different treatments (**a**). Top 20 KEGG enrichment of DAMs (**b**). Sankey diagram of DAMs among various treatments compared with the untreated control (**c**). The significant differentially accumulated alkaloids, flavonoids, amino acids and derivatives responses to Cd (**d**). *Up* upregulated metabolites; *Down* downregulated metabolites; and *NS* non-significant metabolites

To explore whether NPs or ions alter the metabolic response to Cd, we further identified the common metabolites in the Cd-treated tobacco roots and leaves among treatments. KEGG pathway enrichment analysis indicated that several of the top 20 pathways, including arginine and proline metabolism, beta-alanine metabolism, and metabolic pathways, were markedly enriched in tobacco roots and leaves (Figs. 4, 5). Alkaloids, amino acids and their derivatives, and flavonoids were among the most altered metabolites in the Cd-treated seedlings. Specifically, 10 alkaloids, 17 amino acids and their derivatives, and 16 flavonoids showed increased accumulation, whereas 3 alkaloids, 1 amino acid and its derivative, and 3 flavonoids showed decreased accumulation in roots; While 2 alkaloids, 5 amino acids and their derivatives, and 13 flavonoids showed increased accumulation, whereas 2 alkaloids and 1 flavonoid showed decreased accumulation in the leaves of the Cd-treated seedlings (Figs. 4, 5, Additional file 4: Table S3 and Additional file 5: Table S4). Surprisingly, we found that NPs or ions recovered the Cd-responsive metabolites to normal levels in the Cd-treated tobacco roots and leaves (Figs. 4, 5, Additional file 4: Table S3 and Additional file 5: Table S4). For example, Fe_3_O_4_ NPs restored 75 DAMs (including 8 alkaloids, 7 amino acids and their derivatives, 11 flavonoids) in the roots and 53 DAMs (including 4 alkaloids, 1 amino acid and its derivative, 9 flavonoids) in leaves to normal levels. In comparison, FeSO_4_ restored 46 DAMs (including 9 alkaloids, 4 amino acids and their derivatives, 9 flavonoids) in the roots and 51 DAMs (including 3 alkaloids, 3 amino acids and their derivatives, 8 flavonoids) in the leaves of Cd-treated tobacco seedlings to normal levels (Figs. 4, 5). ZnO NPs restored 70 DAMs (including 8 alkaloids, 6 amino acids and their derivatives, 12 flavonoids) in the roots and 48 DAM (including 3 alkaloids, 3 amino acids and their derivatives, 6 flavonoids) in the leaves to normal levels. In comparison, ZnSO_4_ restored 57 DAMs (including 7 alkaloids, 3 amino acids and their derivatives, 9 flavonoids) in the roots and 45 DAMs (including 4 alkaloids, 3 amino acids and their derivatives, 5 flavonoids) in the leaves of Cd-treated tobacco seedlings to normal levels (Figs. 4, 5). These results collectively indicate that NP-reprogrammed DAMs may have great potential in alleviating Cd stress and further support the positive effects of NPs on growth under Cd stress.

### Correlation analyses of DAMs and growth parameters of tobacco seedlings

**Fig. 6:**
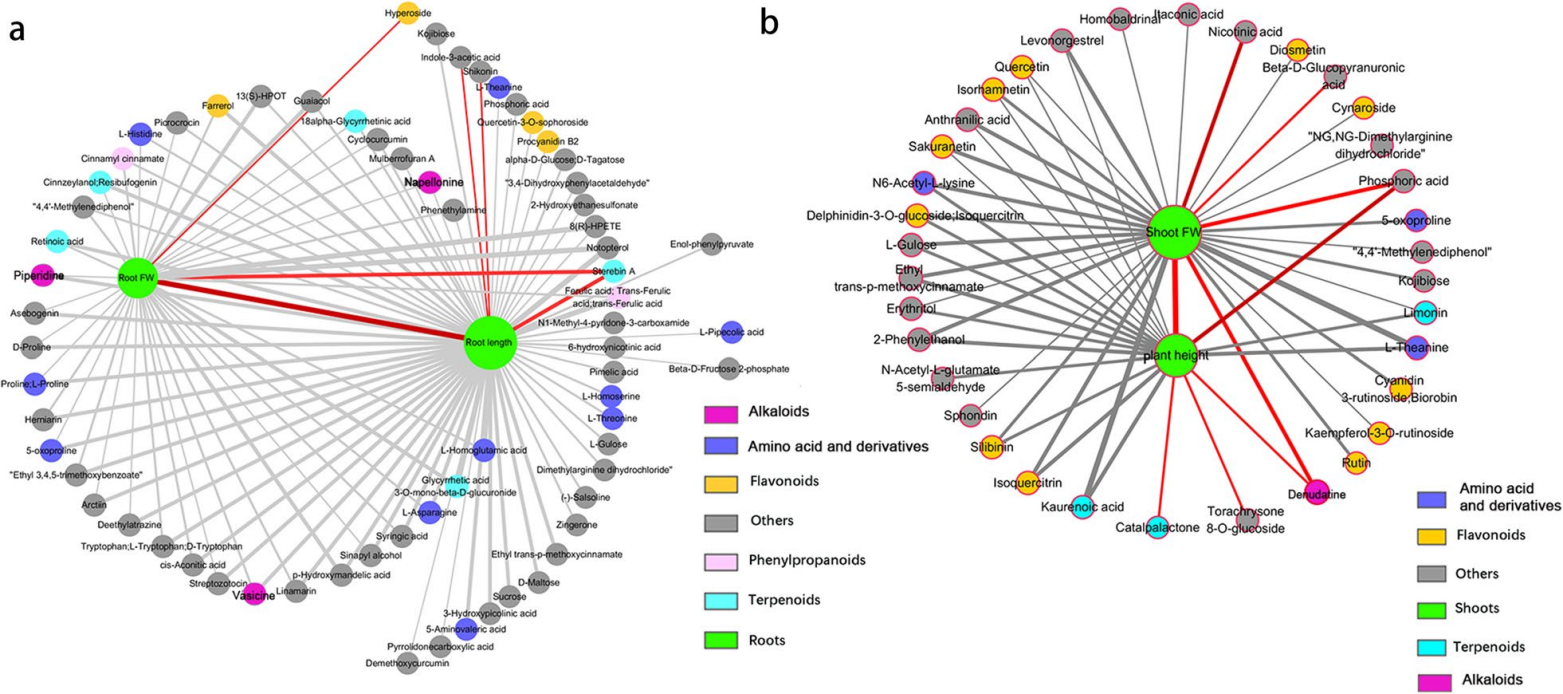
Correlation analysis between growth parameters and differentially accumulated metabolites. The correlation network of DAMs in the roots with the root length and fresh weight (**a**). The correlation network of DAMs in the leaves with the plant height and shoot fresh weight (**b**). The red lines indicate positive correlations, and the gray lines indicate negative correlations. Different colored circles mark the different types of metabolites. *FW* fresh weight

The above results showed that NPs had greater efficiency in alleviating Cd stress, and DAMs profiling might correlate with plant growth. To address this question, we then conducted Pearson correlation coefficient analyses between the mean values of DAMs and growth parameters (including the plant height, shoot FW, root length, and root FW). In the roots, a diterpenoid (sterebin A), phytohormone (indole-3-acetic acid), and quinone (shikonin) were positively correlated with the root length, whereas 3 alkaloids (napellonine, piperidine, and vasicine), 10 amino acids and their derivatives (including L-theanine, L-threonine, proline), 4 terpenoids (including cinnzeylanol, retinoic acid), and 2 phenylpropanoids (cinnamyl cinnamate and ferulic acid) negatively correlated with the root length (Fig. 6a, Additional file 6: Table S5). A diterpenoid (sterebin A) and a flavonoid (hyperoside) were positively correlated with the root FW, whereas 5 amino acids and their derivatives (including proline, L-histidine, L-asparagine), 2 diterpenoids (cinnzeylanol and retinoic acid), a flavonoid (farrerol), and 2 phenylpropanoids (cinnamyl cinnamate and ferulic acid) negatively correlated with the root FW (Fig. 6a, Additional file 6: Table S5).

An organic acid (phosphoric acid) and an alkaloid (denudatine) were positively correlated with the plant height and shoot FW in the leaves. In addition, a sesquiterpenoid (catalpalactone) and miscellaneous compound (torachrysone 8-O-glucoside) showed a positive correlation with the plant height, while nicotinic acid showed a positive correlation with the shoot FW. However, 2 amino acids and their derivatives (N6-acetyl-l-lysine, l-theanine), 6 flavonoids (including quercetin, isorhamnetin, isoquercitrin), and 2 terpenoids (kaurenoic acid and limonin) showed negative correlations with both the plant height and shoot FW (Fig. 6b, Additional file 7: Table S6). In addition, 5-oxoproline, 5 flavonoids (including kaempferol-3-*O*-rutinoside, rutin, cynaroside), a diterpenoid (kaurenoic acid), and other metabolites showed negative correlations with the shoot FW (Fig. 6b, Additional file 7: Table S6).

Further analysis revealed that several DAMs in the roots and leaves correlated with plant growth. As shown in Fig. 6, 2 amino acids and their derivatives (5-oxoproline and l-theanine), a carbohydrate (l-gulose), cinnamic acid and derivative (ethyl trans-p-methoxycinnamate), carboxylic acid and derivative (NG, NG-dimethylarginine dihydrochloride), fatty acyl (kojibiose), and phenol (4,4′-methylenediphenol), showed negative correlations with plant growth parameters. In addition, phosphoric acid in the roots negatively correlated with the root length, whereas phosphoric acid in the leaves positively correlated with plant height and shoot FW (Fig. 6a, b, Additional file 8: Table S7). These results imply that changed metabolite profiling is indeed responsible for plant growth. The comparison of varied DAMs among different treatments is described in detail below.

### Root and leaf metabolic profiling under cadmium stress

Cd toxicity resulted in more metabolites in the roots (88%), but it suppressed higher metabolites in the leaves (29%) of Cd-treated tobacco seedlings (Fig. 3 and Additional file 1: Fig. S6, Additional file 2: Table S1 and Additional file 3: Table S2). In the roots, Cd treatment increased metabolites, including10 alkaloids, 17 amino acids and their derivatives, 16 flavonoids, 4 phenylpropanoids, and 11 phenols (Fig. 3, Additional file 2: Table S1), whereas it reduced metabolites including 3 alkaloids, beta-alanine, 3 flavonoids, and sterebin A (Fig. 3, Additional file 2: Table S1). In addition, Cd stress increased a phytohormone, N-(-)-jasmonoyl)-S-isoleucine, and 5 carbohydrates, including d-glucose 6-phosphate, sucrose, and D-maltose (Fig. 3, Additional file 2: Table S1). By contrast, it decreased indole-3-acetic acid, a critical phytohormone that regulates plant growth and development, and maltotetraose (Fig. 3, Additional file 2: Table S1).

Cd treatment increased metabolites in the leaves, including 2 alkaloids, 5 amino acids and their derivatives, 13 flavonoids, and 2 phenylpropanoids (Fig. 3, Additional file 3: Table S2). By comparison, it decreased metabolites, including 2 alkaloids, 1 flavonoid, 2 phenylpropanoids, and 2 phenols (Fig. 3, Additional file 3: Table S2). Furthermore, Cd stress increased 2 carbohydrates, l-gulose and sucrose, and a phytohormone, (+)-abscisic acid (Fig. 3, Additional file 3: Table S2); By contrast, it decreased nicotinic acid accumulation (Fig. 3, Additional file 3: Table S2).

Venn diagram analysis showed that Cd stress increased 18 metabolites (8.7%), including 4 amino acids and their derivatives, 2 carbohydrates (l-gulose and sucrose), 3 flavonoids (cynaroside, naringenin chalcone, and quercetin), a phenylpropanoid (cinnamyl cinnamate), in both the roots and leaves of Cd-treated seedlings. Whereas it increased 2 metabolites (1%), a flavonoid, farrerol, and an organic acid, phosphoric acid in tobacco roots but decreased in tobacco leaves compared with the control (Additional file 1: Fig. S6, Additional file 2: Table S1 and Additional file 3: Table S2). These results suggest that Cd stress induces different metabolome profiles in the roots and leaves, indicating a distinct mechanistic response to Cd in tobacco roots and leaves.

### Effects of Fe_3_O_4_ NPs on root and leaf metabolic profiling

Fe_3_O_4_ NPs increased 16 metabolites, including 1 alkaloid, 2 flavonoids, and 2 phenylpropanoids, whereas they reduced 24 metabolites, including 3 alkaloids, 3 flavonoids, and a phenylpropanoid, in the roots (Fig. 3, Additional file 2: Table S1). In the leaves, 26 metabolites, including 5 alkaloids, 7 amino acids and their derivatives, and 3 flavonoids, showed increased levels, whereas 19 metabolites, including 2 alkaloids, 2 flavonoids, and a phenylpropanoid, showed decreased levels compared with the control (Fig. 3, Additional file 3: Table S2). Venn diagram analysis showed that most of the DAMs were unique in the roots and leaves of Fe_3_O_4_ NP-exposed seedlings. Fe_3_O_4_ NPs increased 15 (17.9%) in roots and 26 DAMs (31%) in leaves, whereas they decreased 24 (28.6%) and 18 DAM (21.4%) accumulation levels in the roots and leaves, respectively. Furthermore, Fe_3_O_4_ NPs increased a flavonoid, farrerol in the roots but decreased in the leaves (Additional file 1: Fig. S7a).

FeSO_4_ increased 26 metabolites, including 3 alkaloids, 7 flavonoids, and 1 phenylpropanoid, whereas it decreased 14 metabolites, including 2 alkaloids, 2 amino acids and their derivatives, and 2 phytohormones, in the roots (Fig. 3, Additional file 2: Table S1). In addition, FeSO_4_ increased 26 metabolites, including 2 alkaloids, 2 amino acids and their derivatives, 4 flavonoids, whereas it reduced 13 metabolites, including a flavonoid, 2 phenols, a carbohydrate, in the leaves (Fig. 3, Additional file 3: Table S2). Venn diagram analysis showed FeSO_4_ increased 25 DAMs (32.1%) in the roots and 25 DAMs (32.1%) in the leaves, whereas it decreased 14 DAMs (17.9%) in the roots and 13 DAMs (16.7%) in the leaves. In addition, it increased only one metabolite, arachidonic acid, in both the roots and leaves (Additional file 1: Fig. S7b).

### Effects of Fe_3_O_4_ NPs on the root and leaf metabolomes under Cd stress

Compared with Cd treatment alone, Fe_3_O_4_ NPs increased 10 metabolites, including 3 alkaloids, 2 amino acids and their derivatives, and a terpenoid, while they decreased 27 metabolites, including 6 alkaloids, an amino acid, and 3 flavonoids in the roots (Additional file 2: Table S1). In addition, Fe_3_O_4_ NPs increased 30 metabolites, including 3 alkaloids, 7 amino acids and their derivatives, and 3 flavonoids, whereas they decreased 18 metabolites, including an alkaloid, 4 flavonoids, and 2 phenylpropanoids, in the leaves of Cd-treated seedlings compared with Cd-treated seedlings (Additional file 3: Table S2). Venn diagram analysis showed that Fe_3_O_4_ NPs increased the levels of 10 (11.8%) metabolites in the roots (Additional file 1: Fig. S8a, Additional file 2: Table S1) and 30 (35.3%) metabolites in the leaves (Additional file 1: Fig. S8a, Additional file 3: Table S2) but decreased 27 (31.8%) metabolites in the roots (Additional file 1: Fig. S8a, Additional file 2: Table S1) and 18 (21.2%) metabolites in the leaves of Cd-treated tobacco seedlings (Additional file 1: Fig. S8a, Additional file 3: Table S2). These results indicated that Fe_3_O_4_ NPs reprogram different metabolic profiling responses under Cd stress in the roots and leaves.

**Fig. 7:**
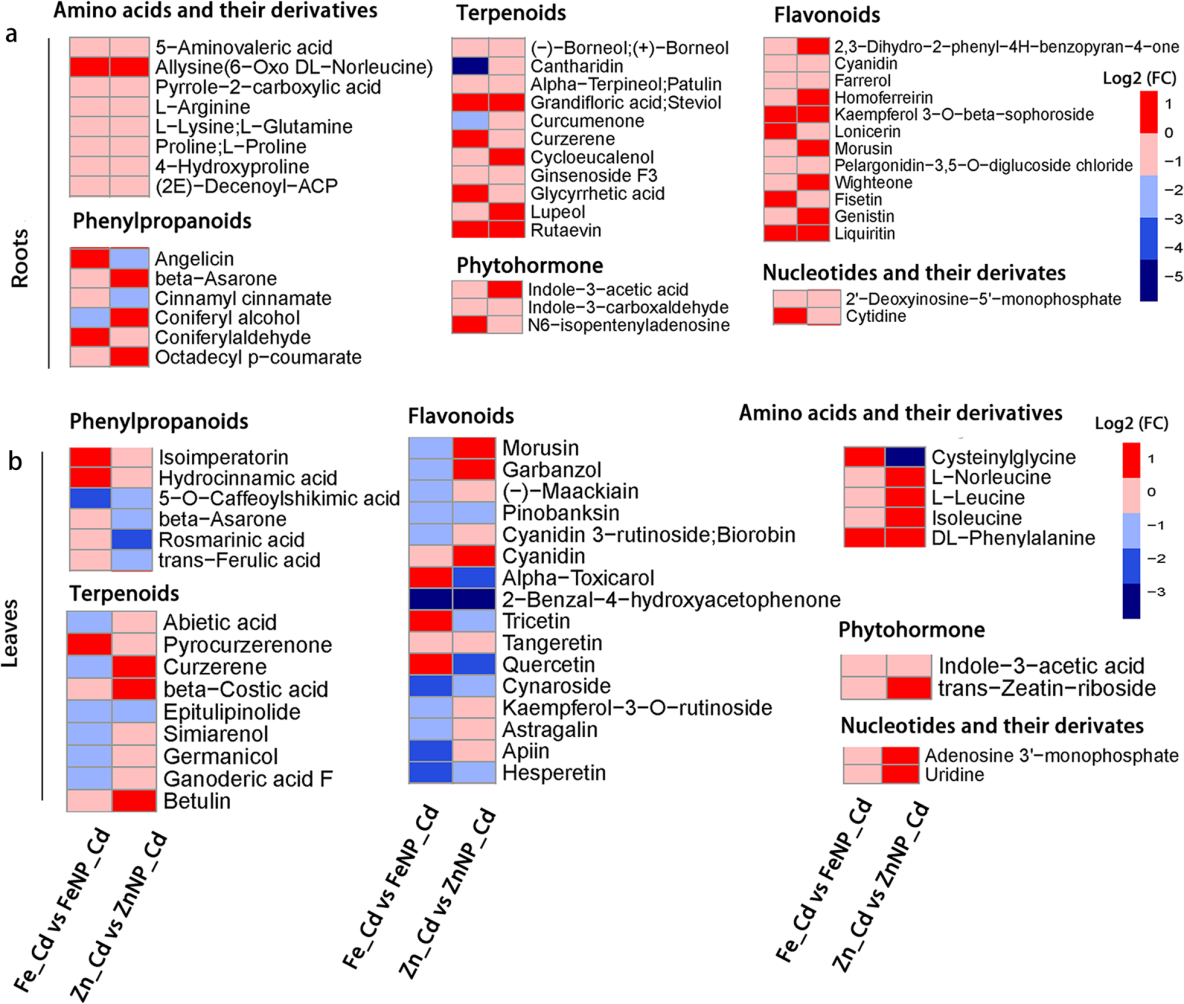
Comparative analyses of differentially accumulated metabolites between NPs and ions under Cd stress. Heat map of important DAM (amino acids, terpenoids, flavonoids, phenylpropanoids, phytohormones, nucleotides and their derivatives) levels in the roots (**a**). Heat map of important DAM (phenylpropanoids, terpenoids, flavonoids, amino acids, phytohormones, nucleotides and their derivatives) levels in the leaves (**b**)

FeSO_4_ increased 37 metabolites, including 5 alkaloids, 2 amino acids and their derivatives, and 6 flavonoids, whereas it decreased 9 metabolites, including 3 alkaloids, a phenylpropanoid, and a terpenoid, in tobacco roots (Additional file 2: Table S1). Additionally, FeSO_4_ increased 53 metabolites, including 5 alkaloids, 2 amino acids and their derivatives, and 8 flavonoids, whereas it reduced 19 metabolites, including an alkaloid, 2 flavonoids, and 3 phenylpropanoids, in the leaves (Additional file 3: Table S2). Venn diagram analysis showed that FeSO_4_ increased the accumulation levels of 37 (31.9%) in the roots and 53 DAMs (45.7%) in the leaves, while it decreased the accumulation levels of 9 (7.8%) and 19 DAMs (16.4%) in the roots and leaves, respectively (Additional file 1: Fig. S8b, Additional file 2: Table S1 and Additional file 3: Table S2). Furthermore, a flavonoid, homoferreirin, showed an increased accumulation level in the roots and leaves. By contrast, it reduced a phenylpropanoid, 3-methoxy-4,5-methylenedioxycinnamaldehyde, in both the roots and leaves (Additional file 1: Fig. S8b, Additional file 2: Table S1 and Additional file 3: Table S2).

### Effects of ZnO NPs on root and leaf metabolic profiling

ZnO NPs increased 30 metabolites, including 3 alkaloids, 9 flavonoids, and 3 phenylpropanoids, whereas they reduced 22 metabolites, including an alkaloid, 5 amino acids and their derivatives, and 3 flavonoids, in tobacco roots (Fig. 3a, Additional file 2: Table S1). In addition, ZnO NPs increased 12 metabolites, including 2 alkaloids, 4 amino acids and their derivatives, and a flavonoid, whereas they reduced 17 metabolites, including 2 alkaloids, 5 flavonoids, and 2 nucleotides and their derivates, in tobacco leaves (Fig. 3a, Additional file 3: Table S2). Venn diagram analysis showed that ZnO NPs increased 30 (37%) in the roots and 12 DAM (14.8%) in the leaves, while they decreased 22 DAMs (27.2%) and 17 DAMs (21%) levels in the roots and leaves, respectively, compared with the control (Additional file 1: Fig. S9a, Additional file 2: Table S1 and Additional file 3: Table S2).

ZnSO_4_ increased 34 metabolites, including 3 alkaloids and 6 flavonoids, whereas it reduced 26 metabolites, including 7 alkaloids, 2 amino acids and their derivatives, and 4 flavonoids, in the roots (Fig. 3a, Additional file 2: Table S1). Meanwhile, ZnSO_4_ increased 16 metabolites, including an alkaloid, 4 amino acids and their derivatives, and a flavonoid, whereas it reduced 17 metabolites, including an alkaloid, 6 flavonoids, and a phytohormone (1-naphthylacetic acid), showed decreased levels in the leaves (Fig. 3a, Additional file 3: Table S2). Venn diagram analysis showed that ZnSO_4_ increased 34 (36.6%) in the roots and 16 DAMs (17.2%) in the leaves, while it decreased 26 (28%) in the roots and 17 DAMs (18.3%) in the leaves, compared with the control (Additional file 1: Fig. S9b).

### Effects of ZnO NPs on root and leaf metabolomes under Cd stress

Compared with Cd treatment alone, ZnO NPs increased 35 metabolites, including 2 alkaloids, 3 amino acids and their derivatives, and 5 flavonoids, while it decreased 15 metabolites, including 2 alkaloids, 2 flavonoids, and a phenol, in the roots (Additional file 2: Table S1). In addition, ZnO NPs increased 32 metabolites, including 8 amino acids and their derivatives, 2 flavonoids, and 2 nucleotides and their derivatives. In contrast, they reduced 15 metabolites, including 3 alkaloids, 2 amino acids and their derivatives, and 2 flavonoids, in the leaves of Cd-treated tobacco (Additional file 3: Table S2). Venn diagram analysis showed that ZnO NPs increased 35 (36.4%) metabolites in tobacco roots (Additional file 1: Fig. S10a, Additional file 2: Table S1) and 32 (33.3%) metabolites in tobacco leaves (Additional file 1: Fig. S10a, Additional file 3: Table S2), whereas ZnO NPs decreased 15 (15.6%) metabolites in the roots (Additional file 1: Fig. S10a, Additional file 2: Table S1) and 15 (15.6%) metabolites in the leaves of Cd-treated tobacco (Additional file 1: Fig. S10a, Additional file 2: Table S1). Only one metabolite, an alkaloid [3-(carboxymethylamino) propanoic acid], was upregulated in the roots but reduced in the leaves of Cd-treated tobacco (Additional file 1: Fig. S10, Additional file 2: Table S1 and Additional file 3: Table S2).

ZnSO_4_ increased 56 metabolites, including 8 alkaloids, 8 amino acids and their derivatives, and 3 flavonoids, while it decreased 12 metabolites, including 2 alkaloids, 3 flavonoids, and a phytohormone, in the roots (Additional file 2: Table S1). In addition, ZnSO_4_ increased 45 metabolites, including 2 alkaloids, 5 amino acids and their derivatives, 5 flavonoids, and 5 phenols, whereas it reduced19 metabolites, including an alkaloid, 2 flavonoids, and a phenol, in the leaves of Cd-treated seedlings (Additional file 3: Table S2). Venn diagram analysis revealed that ZnSO_4_ increased 56 DAMs (43.7%) accumulation levels in the roots and 45 DAMs (35.1%) accumulation levels in the leaves. In comparison, it decreased 12 DAMs (9.4%) accumulation levels in the roots and 19 DAMs (14.9%) accumulation levels in the leaves (Additional file 1: Fig. S10b, Additional file 2: Table S1 and Additional file 3: Table S2). Furthermore, 3 metabolites, including beta-D-fructose 2-phosphate, l-acetylcarnitine, and l-citruline, showed an increased accumulation level in the roots and leaves. By contrast, cytidine 5′-monophosphate showed a decreased accumulation level in the roots and decreased in the leaves of Cd-treated seedlings (Additional file 1: Fig. S10b, Additional file 2: Table S1 and Additional file 3: Table S2).

### Comparative analyses of DAMs between Fe_3_O_4_ and ZnO NP-treated seedlings under normal conditions and Cd stress

Under untreated control conditions, both Fe_3_O_4_ NPs and ZnO NPs increased 6 (4%) metabolites, including L-arginine, l-threonine, and quercetin, whereas they reduced 9 (6%) metabolites, including glycyrrhetinic acid, adenosine 3′-monophosphate, and uridine 5'-monophosphate, in the roots and/or leaves (Additional file 1: Fig. S11, Additional file 2: Table S1 and Additional file 3: Table S2).

Under Cd stress conditions, Fe_3_O_4_ NPs and ZnO NPs increased 13 (8.3%) metabolites, including 5 amino acids and their derivatives (e.g., isoleucine, l-leucine, l-phenylalanine), 2 flavonoids (7-hydroxyflavone and farrerol), 6-aminocaproic acid, and cytidine 5′-monophosphate; however, they decreased 9 (5.7%) metabolites, including 2 alkaloids [3-(carboxymethylamino) propanoic acid and jervine], and lupeol D-maltose, in the roots and/or leaves of Cd-treated tobacco seedlings (Additional file 1: Fig. S12, Additional file 2: Table S1 and Additional file 3: Table S2). In addition, Fe_3_O_4_ NPs increased the level, but ZnO NPs decreased the level of 2'-deoxyinosine-5'-monophosphate, while ZnO NPs increased the level, but Fe_3_O_4_ NPs decreased the level of (13E)-11a-hydroxy-9,15-dioxoprost-13-enoic acid in the Cd-treated tobacco roots and/or leaves (Additional file 1: Fig. S12, Additional file 2: Table S1 and Additional file 3: Table S2). The above results suggest that the two NPs mediate Cd tolerance through similar and distinct mechanisms.

### Comparative analyses of DAMs between FeSO_4_- and ZnSO_4_-treated seedlings under normal conditions and Cd stress

Under untreated control conditions, FeSO_4_ and ZnSO_4_ increased 6 (3.7%) metabolites, including lupenone, nicotinic acid adenine dinucleotide, and synephrine, whereas they decreased 4 (2.5%) metabolites, including cantharidin, deethylatrazine, glycyrrhetinic acid, and L-glutamic acid, in the roots and/or leaves (Additional file 1: Fig. S13, Additional file 2: Table S1 and Additional file 3: Table S2).

Under Cd stress conditions, FeSO_4_ and ZnSO_4_ increased 21 (9.8%) metabolites, including 2 alkaloids (cinchonine, methylisopelletierine), 4 amino acids and their derivatives (e.g., l-citruline, l-lysine, and 4-aminobutyric acid), and 2 phenols (5-heneicosylresorcinol and oleocanthal). However, it decreased 4 (1.9%) metabolites, including dihydromyricetin, taraxasterone, and hypotaurine, in the Cd-treated tobacco roots and/or leaves (Additional file 1: Fig. S14, Additional file 2: Table S1 and Additional file 3: Table S2). However, FeSO_4_ increased the level, but ZnSO_4_ decreased 3 metabolites, including guggulsterone E&Z, cytidine 5′-monophosphate, and octadecyl p-coumarate, while ZnSO_4_ increased, but FeSO_4_ decreased the level of 2 metabolites, angelicin and picrasin B, in the Cd-treated tobacco roots and/or leaves (Additional file 1: Fig. S14, Additional file 2: Table S1 and Additional file 3: Table S2). These results indicate that the two ions might also modulate the Cd response through similar and distinct mechanisms.

### Comparative analyses of DAMs between Fe_3_O_4_ NP- and FeSO_4_-treated seedlings under normal conditions and Cd stress

Under untreated control conditions, Fe_3_O_4_ NPs and FeSO_4_ increased 7 (4.8%) metabolites, including farrerol, isoalantolactone, and rosmarinine, whereas they decreased 8 (5.5%) metabolites, including 2 diterpenoids (picrasin B, kaurenoic acid), glycyrrhetinic acid, and adenosine 3′-monophosphate, in the roots and/or leaves. In addition, FeSO_4_ increased, but Fe_3_O_4_ NPs decreased the level of officinalisinin I (Additional file 1: Fig. S15, Additional file 2: Table S1 and Additional file 3: Table S2). Under Cd stress conditions, Fe_3_O_4_ NPs and FeSO_4_ increased 7 (3.9%) metabolites, including alpha-d-glucose, aconitine, and cytidine 5'-monophosphate. In contrast, they decreased the levels of 8 (4.4%) metabolites, including 3 alkaloids [3-(carboxymethylamino) propanoic acid, anacrotine, piperidine], dihydromyricetin, and erythritol, in the Cd-treated tobacco roots and/or leaves (Fig. 7 and Additional file 1: Fig. S16, Additional file 2: Table S1 and Additional file 3: Table S2). However, FeSO_4_ increased, but Fe_3_O_4_ NPs decreased 5 metabolites, including apiin, phyllalbine, and herniarin, in the Cd-treated tobacco roots and/or leaves (Fig. 7 and Additional file 1: Fig. S16, Additional file 2: Table S1 and Additional file 3: Table S2).

### Comparative analyses of DAMs between ZnO NP- and ZnSO_4_-treated seedlings under normal conditions and Cd stress

ZnO NPs and ZnSO_4_ increased 8 (5.1%) metabolites, including l-homoserine, L-threonine, and dexamethasone. In contrast, they decreased 8 (5.1%) metabolites, including 3 flavonoids (kaempferol, methyl hesperidin, and hyperoside), 3-carbamyl-1-methylpyridinium 1-methylnicotinamide, and acarbose, in the roots and/or leaves under untreated control conditions (Additional file 1: Fig. S17, Additional file 2: Table S1 and Additional file 3: Table S2). Under Cd stress conditions, both ZnO NPs and ZnSO_4_ increased the levels of 16 (7.9%) metabolites, including 4 amino acids and their derivatives (e.g., DL-alanine, l-theanine, L-citruline), 3 nucleotides and their derivatives (NAD, beta-nicotinamide mononucleotide, and cytidine 5′-monophosphate), and astaxanthin. In contrast, they decreased the levels of 3 (1.5%) metabolites, tacrolimus, 13(S)-HPOT and cedeodarin, in the roots and/or leaves of Cd-treated tobacco seedlings (Fig. 7 and Additional file 1: Fig. S18, Additional file 2: Table S1 and Additional file 3: Table S2). However, ZnO NPs increased the level, but ZnSO_4_ decreased (13E)-11a-hydroxy-9,15-dioxoprost-13-enoic acid. Additionally, ZnSO_4_ increased, but ZnO NPs decreased 2 metabolites, d-proline and l-proline, in the Cd-treated tobacco roots and/or leaves (Fig. 7 and Additional file 1: Fig. S18, Additional file 2: Table S1 and Additional file 3: Table S2). The above results suggested that NPs and ions might largely modulate the Cd response through distinct mechanisms.

### Fe_3_O_4_ or ZnO NPs altered critical metabolite pathway under Cd stress

**Fig. 8:**
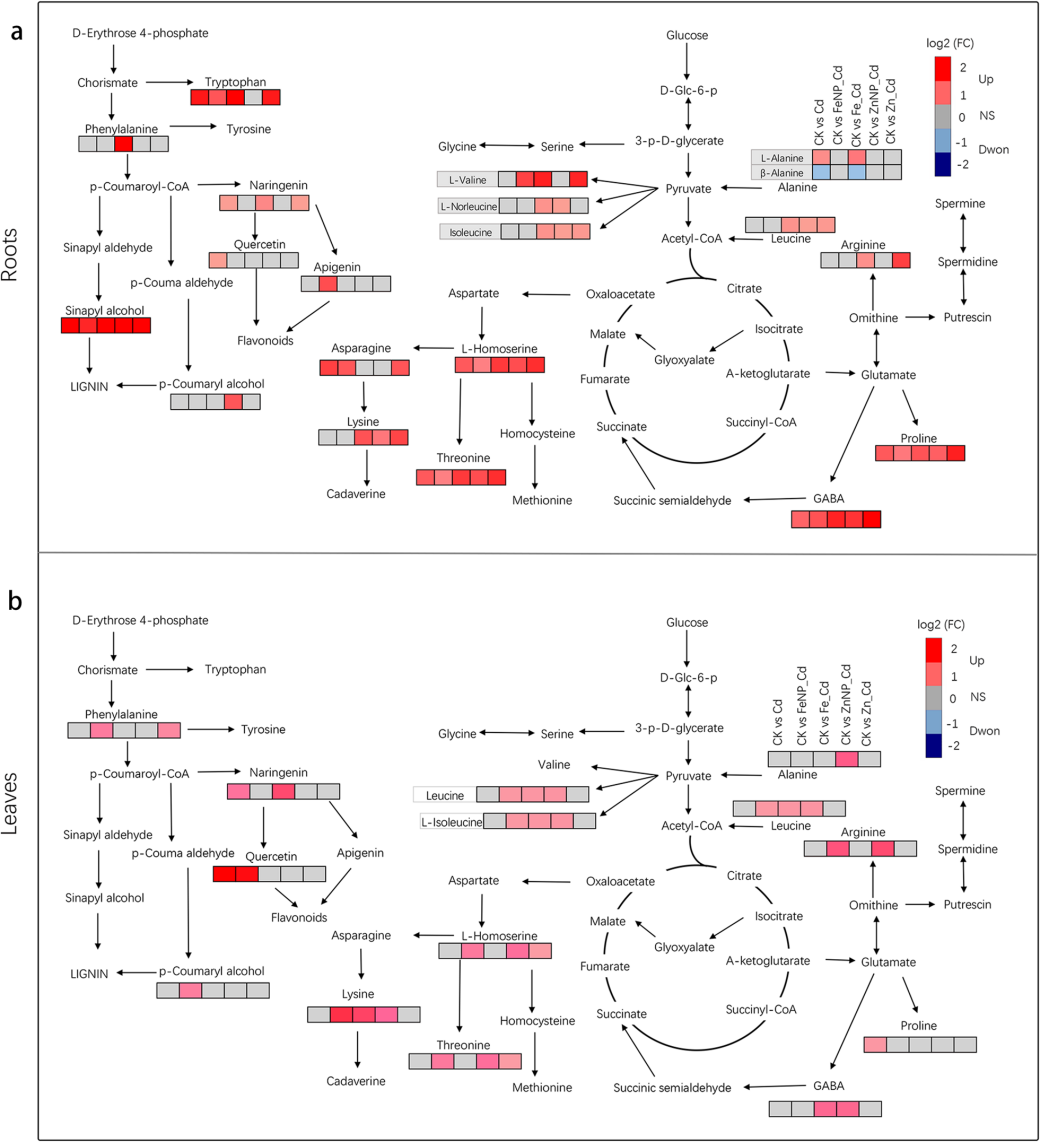
Amino acid-related metabolic pathway response to Cd stress. The amino acid metabolism pathway and related metabolites accumulation levels in the roots (**a**) and leaves (**b**) compared with the untreated control. Red and blue boxes present metabolites that showed higher or lower abundance than the untreated control, and gray boxes indicate that metabolites are unchanged

The above results indicated that Fe_3_O_4_ and ZnO NPs showed more efficiency in facilitating tobacco growth than ions under Cd stress (Figs. 1, 3, 4, 5). Furthermore, the common Cd-induced DAMs were significantly enriched in the pathway of amino acids metabolism, flavone and flavonol biosynthesis, secondary metabolite biosynthesis, nicotinate and nicotinamide metabolism, indicating that these two NPs reprogram carbon/nitrogen metabolism and secondary metabolism (Figs. 3, 4, 5). Thus, we further analyzed these metabolites involved in amino acid, nicotinate and nicotinamide metabolism pathway in response to Cd stress with or without exposure to Fe_3_O_4_ or ZnO NPs as well as ions. Specifically, Cd stress increased 7 amino acids and their derivatives, including l-alanine, tryptophan, and 4-aminobutyric acid, but decreased beta-alanine in tobacco roots (Fig. 8a, Additional file 2: Table S1). Interestingly, Fe_3_O_4_ NPs, ZnO NPs, and ions increased 4 amino acids (proline, 4-aminobutyric acid, l-homoserine, l-threonine) and sinapyl alcohol in the Cd-treated tobacco roots compared with untreated control (Fig. 8a, Additional file 2: Table S1). However, only proline showed increased accumulation in the leaves, while the other amino acids were not affected after Cd treatment (Fig. 8b, Additional file 3: Table S2). Both Fe_3_O_4_ and ZnO NPs showed higher accumulation levels of 7 amino acids, including leucine, arginine, and threonine, in the Cd-treated tobacco leaves than untreated control (Fig. 8b, Additional file 2: Table S1 and Additional file 4: Table S3). Furthermore, Fe_3_O_4_ NPs increased phenylalanine, p-coumaryl alcohol, and quercetin levels, while ZnO NPs increased alanine and 4-aminobutyric acid levels in the Cd-treated tobacco leaves (Fig. 8b, Additional file 3: Table S2 and Additional file 5: Table S4). These results indicated that Fe_3_O_4_ or ZnO NPs improve seedling growth by reprogramming amino acid metabolism in the roots and leaves under Cd stress.

**Fig. 9:**
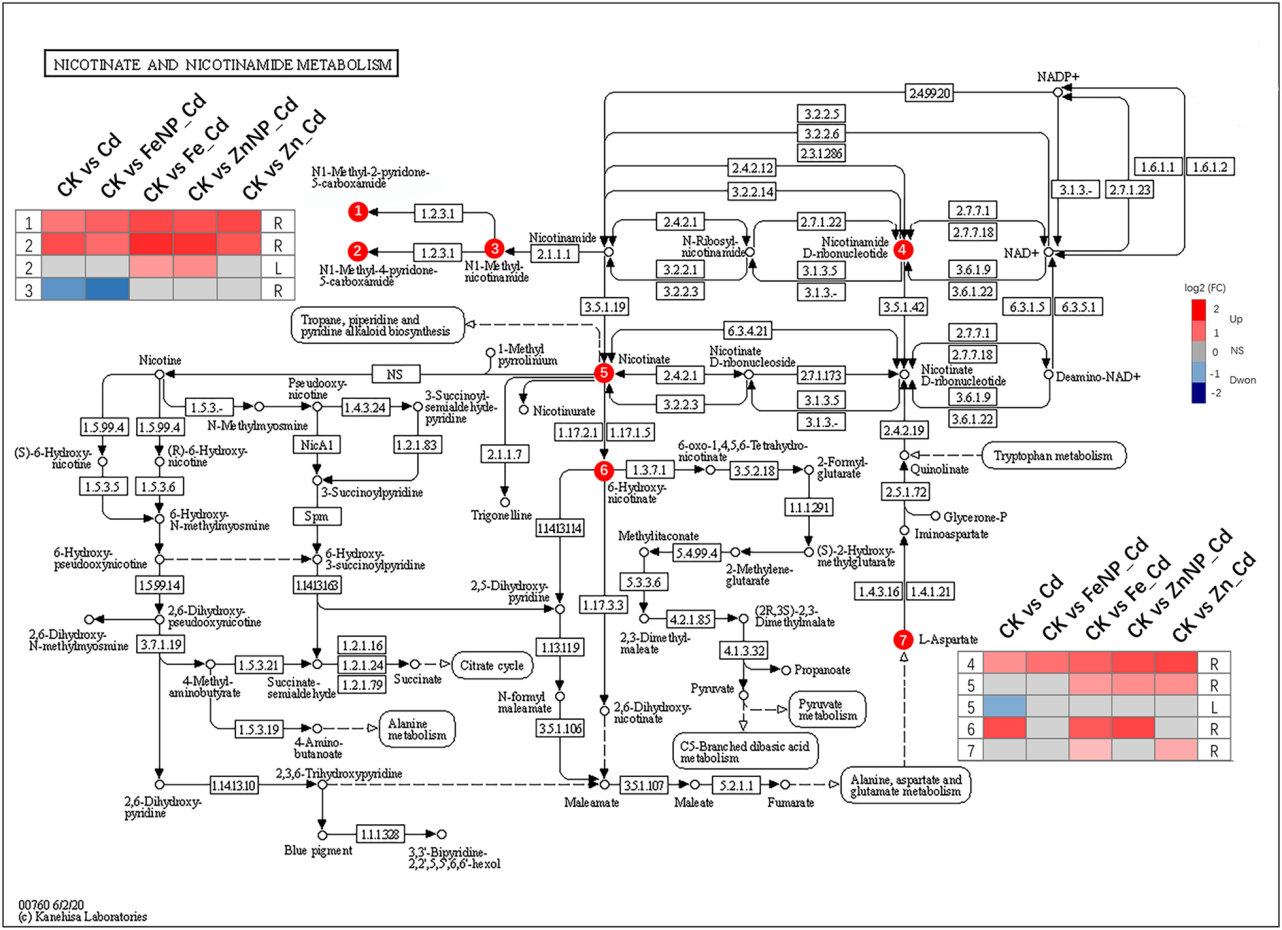
KEGG pathway analysis of nicotinate and nicotinamide metabolism responses to Cd stress. Compared with the untreated control, the nicotinamide metabolism pathways and related metabolite accumulation levels in the roots and leaves. Red and blue boxes present metabolites that showed higher or lower abundance than the untreated control, and gray boxes indicate that metabolites are unchanged. *R* roots; *L* leaves

Nicotinate and nicotinamide metabolism play roles in plant response to stress [46]. We thus mapped the Cd-induced DAMs involved in the nicotinate and nicotinamide metabolism pathway (map00760) to the KEGG pathway database [47]. In the roots, Cd stress increased N1-methyl-2-pyridone-5-carboxamide (C05842), N1-methyl-4-pyridone-5-carboxamide (C05843), beta-nicotinamide mononucleotide (C00455) and 6-hydroxynicotinic acid (C01020), but decreased N1-methylnicotinamide (C02918) (Fig. 9, Additional file 2: Table S1). Similarly, NPs and ions increased the levels of N1-methyl-2-pyridone-5-carboxamide (C05842), N1-methyl-4-pyridone-5-carboxamide (C05843), and beta-nicotinamide mononucleotide (C00455) in the Cd-treated tobacco roots compared with the untreated control (Fig. 9, Additional file 2: Table S1). Furthermore, FeSO_4_ increased nicotinate (C00253), 6-hydroxynicotinic acid (C01020), and L-aspartic acid (C00049) levels, whereas Fe_3_O_4_ NPs decreased N1-methylnicotinamide (C02918) levels. ZnO NPs increased nicotinate (C00253) and 6-hydroxynicotinic acid (C01020) levels, while ZnSO_4_ increased nicotinate (C00253) and L-aspartic acid (C00049) levels in the Cd-treated tobacco roots compared with the untreated control (Fig. 9, Additional file 2: Table S1). In the leaves, FeSO_4_ and ZnO NPs increased N1-methyl-4-pyridone-5-carboxamide (C05843) levels under Cd stress, whereas Fe_3_O_4_ NPs or ZnSO_4_ did not affect its accumulation levels in the Cd-treated leaves compared with the untreated control (Fig. 9, Additional file 3: Table S2). In addition, the accumulation of nicotinate (C00253) showed a decrease in the Cd-treated leaves, whereas NPs did not affect its levels in the leaves of Cd-treated tobacco seedlings compared with the untreated control (Fig. 9, Additional file 3: Table S2). These results indicated that NPs or ions modulate nicotinate and nicotinamide metabolism to regulate plant response to Cd stress.

## Discussion

Cd toxicity repressed tobacco growth, as indicated by the plant height, shoot FW, root length, and root FW (Fig. 1). However, foliar exposure to Fe_3_O_4_ or ZnO NPs alleviated inhibitory effects of Cd stress, and NPs were more efficient in improving seedling growth than ions (Fig. 1). Fe_3_O_4_-based NPs effectively alleviate the effects of Cd on growth and yield parameters [22] and relieve Cd-induced growth inhibition by modulating the antioxidant capacity [23, 24]. Furthermore, ZnO NPs can alleviate Cd toxicity in maize [33]. Our results further convinced the facilitating effects of these NPs. In addition, the concentration of ions released from NPs was far below the concentration of applied ions (Additional file 1: Fig. S2), indicating that the effects of NPs on plant growth might not be attributed to the ions released from NPs but rather from themselves. These results suggest that NPs and ions might regulate the plant response to Cd toxicity through distinct mechanisms.

The mineral element accumulation levels, distribution, and balance play essential roles in maintaining plant growth [48]. We found that Cd perturbed the mineral balance in the roots and leaves (Fig. 2). Furthermore, Cd toxicity is often accompanied by severe oxidative damage, which seriously represses plant growth [49]. In this study, Cd stress markedly reduced Fe, Mn and Zn in the roots and Mn and Cu in the leaves (Fig. 2). These elements are critical components of superoxide dismutase (SOD), such as FeSOD, MnSOD, and Cu/ZnSOD, which are responsible for the redox status of cells [50]. The decreased contents of Fe, Mn, Cu and Zn in Cd-treated seedlings might reduce enzyme activity, implying that Cd stress might induce oxidative damage in tobacco seedlings, thereby repressing plant growth. Interestingly, Fe_3_O_4_ and ZnO NPs increased some beneficial nutrients (Zn, K, Mn, Cu, Mg) in the roots and/or leaves, and these increased macro- and micro-elements were conducive to plant growth, thus improving growth under Cd stress.

Plants adopt efficient strategies, including molecular regulation, signal transduction, and alteration of metabolic pathways, to resist Cd toxicity [42, 49]. Cd stress induced higher metabolites in the roots than in the leaves (Fig. 3, Additional file 2: Table S1 and Additional file 3: Table S2). Amino acids, such as l-theanine and l-proline, are compatible substances that protect plants against stress [51]. Cd indeed markedly increased l-theanine, l-histidine, and l-proline levels in the roots and leaves (Additional file 1: Fig. S6, Additional file 2: Table S1 and Additional file 3: Table S2). Chen et al. [51] demonstrated that theanine plants confer salt stress tolerance by regulating redox homeostasis. We also found that l-theanine was induced by Cd stress, and its manner of affecting Cd stress is still elusive. In addition, beta-alanine, a nonproteinogenic amino acid, was significantly decreased under Cd stress, which was inconsistent with the previous study [49]. Furthermore, indole-3-acetic acid, a critical phytohormone that regulates plant growth and development [52, 53], showed decreased accumulation in the Cd-treated roots, thereby repressing root growth under Cd toxicity (Fig. 3). We also found that Cd stress increased several important DAMs, including 2 carbohydrates, 3 flavonoids, and a phenylpropanoid, showed increased accumulation in both the roots and leaves, whereas it increased farrerol and phosphoric acid in the roots but decreased in the leaves (Additional file 1: Fig. S6, Additional file 2: Table S1 and Additional file 3: Table S2). These results indicated that Cd stress-induced metabolome profiling conferred a distinct mechanistic response to Cd in the roots and leaves.

Studies have been performed on Fe_3_O_4_ NPs and bulked Fe_3_O_4_ as well as ZnO NPs and bulked ZnO [21, 28], and the effects of NPs and ions on plants are different [27, 29, 30]. Our study found that the ion content released from Fe_3_O_4_ or ZnO NPs was far below the concentration of applied ions, and their effects on the plant might be attributed to themselves. Under untreated control conditions, Fe_3_O_4_ NPs and FeSO_4_ increased the levels of 7 metabolites but decreased 8 metabolites in the roots and/or leaves (Additional file 1: Fig. S15). Under Cd stress conditions, Fe_3_O_4_ NPs and FeSO_4_ increased the levels of 7 metabolites but decreased 8 metabolites in the roots and/or leaves of Cd-treated tobacco seedlings (Fig. 7 and Additional file 1: Fig. S16). However, Fe_3_O_4_ NPs decreased, but FeSO_4_ increased 5 metabolites in the Cd-treated tobacco roots and/or leaves (Fig. 7 and Additional file 1: Fig. S16).

Likewise, under untreated control conditions, ZnO NPs and ZnSO_4_ increased the levels of 8 metabolites but decreased 8 metabolites in the roots and/or leaves (Additional file 1: Fig. S17). Under Cd stress conditions, ZnO NPs and ZnSO_4_ increased the levels of 16 metabolites but decreased 3 metabolites in the roots and/or leaves of Cd-treated seedlings (Fig. 7 and Additional file 1: Fig. S18). However, ZnSO_4_ increased, but ZnO NPs decreased the levels of 2 metabolites in the Cd-treated roots and/or leaves (Fig. 7 and Additional file 1: Fig. S18). These results provided compelling evidence that NPs and ions might largely modulate the Cd response through distinct mechanisms.

Both Fe and Zn are indispensable elements for a set of physiological and biochemical processes [31, 54]. Fe_3_O_4_ and ZnO NPs play pivotal roles in alleviating Cd toxicity [22, 23, 33]. Under untreated control conditions, both Fe_3_O_4_ and ZnO NPs increased the accumulation of 6 metabolites but decreased 9 metabolites in the roots and/or leaves (Additional file 1: Fig. S11). Among them, decreased adenosine 3'-monophosphate (AMP) and uridine 5'-monophosphate (UMP) might act as signaling molecules in response to stress [55]. Under Cd stress conditions, both Fe_3_O_4_ and ZnO NPs increased 13 metabolites, including l-phenylalanine, alpha-d-glucose, and cytidine 5′-monophosphate (CMP). These primary metabolites promote carbon/nitrogen metabolism, thus enhancing stress tolerance [32]. On the other hand, they decreased 8 metabolites in the Cd-treated roots and/or leaves (Additional file 1: Fig. S12). However, (13E)-11a-hydroxy-9,15-dioxoprost-13-enoic acid and 2′-deoxyinosine-5′-monophosphate showed opposite accumulation levels in the roots and/or leaves of foliar-sprayed with Fe_3_O_4_ or ZnO NPs under Cd stress (Additional file 1: Fig. S12). Taken together, these results suggested that these two NPs enhance Cd tolerance through similar and distinct mechanisms.

Primary metabolism, including sugars, amino acids, and nucleic acids, affects plant adaptation to the environment, while secondary metabolites are non-essential but play pleiotropic roles in modulating plant responses to abiotic and biotic stresses [56, 57]. We found that both NPs restored more DAMs to normal levels in the Cd-treated tobacco roots and leaves compared with ions (Figs. 4, 5, Additional file 4: Table S3 and Additional file 5: Table S4). These recovered metabolites primarily included alkaloids, amino acids and derivatives, flavonoids, and phenols. Our correlation analysis demonstrated that many metabolites, including alkaloids, flavonoids, and phenylpropanoids, significantly correlated with plant growth (Fig. 6, Additional file 6: Table S5, Additional file 7: Table S6 and Additional file 8: Table S7). We indeed found that Fe_3_O_4_ and ZnO NPs promoted quicker seedling growth under Cd toxicity than ions (Fig. 1).

Amino acids participate in various processes by acting as intermediate metabolites and protectants in plants [58]. For example, proline, an osmotic adjustment substance, protects the plant against stress [59]. Furthermore, 4-aminobutyric acid (GABA) plays a critical role in the plant response to stress [60]. Indeed, we found that Fe_3_O_4_ or ZnO NPs increased 4 amino acids (proline, GABA, l-homoserine, and l-threonine) and sinapyl alcohol in the roots, while they increased 7 amino acids in the leaves of Cd-treated tobacco seedlings compared with untreated control plants (Fig. 8). Furthermore, we also found that Fe_3_O_4_ or ZnO NPs restored more Cd-induced amino acids to normal levels in Cd-treated roots and/or leaves compared with untreated control plants (Figs. 4, 5). These results indicated that Fe_3_O_4_ or ZnO NPs improve seedling growth via alterations in amino acid metabolism in the roots and leaves under Cd toxicity, which played pivotal roles in balancing plant growth and stress tolerance.

Numerous stimuli induce secondary metabolites, including temperature, salinity, and heavy metal stress [61, 62]. However, plants produce higher concentrations of secondary metabolites at the expense of slowing plant growth and have evolved mechanisms for the trade-off between primary and secondary metabolism [63, 64]. Secondary metabolites, including alkaloids, flavonoids, and nicotinic acid derivatives, play essential roles in the plant response to stress. Furthermore, they were highly induced under heavy metal stress, which confers tolerance to toxic heavy metals [65, 66]. In this study, we found that most of the Cd-induced alkaloids were upregulated in the roots and/or leaves under Cd stress, whereas both Fe_3_O_4_ and ZnO NPs recovered more Cd-induced alkaloids to normal levels in the roots and/or leaves under Cd stress (Figs. 4, 5, Additional file 4: Table S3 and Additional file 5: Table S4). These results suggested that NPs also facilitated plant growth by reprogramming alkaloid metabolism.

As critical secondary metabolites, flavonoids play pivotal roles in mediating abiotic and biotic stress in plants [67]. Previous studies demonstrate that flavonoids negatively regulate auxin transport and repress auxin redistribution, thus affecting plant growth [68, 69]. In this study, we found that most Cd-induced flavonoids were upregulated in the roots and leaves under Cd stress, and the accumulation of IAA was decreased in the Cd-treated roots. However, Fe_3_O_4_ and ZnO NPs recovered more Cd-induced flavonoids to normal levels, similar to amino acids and alkaloids (Figs. 4, 5, Additional file 4: Table S3 and Additional file 5: Table S4).

Nicotinate and nicotinamide metabolism play roles in various processes in plants [46]. Nicotinamide adenine dinucleotide (NAD) acts as an essential coenzyme and is generally active in vigorous cells [70, 71]. We found that nicotinic acid and NAD accumulation decreased under Cd toxicity but showed an increase or insignificant change in the NP- or ion-exposed leaves under Cd stress. Moreover, N1-methyl-2-pyridone-5-carboxamide, N1-methyl-4-pyridone-5-carboxamide, 6-hydroxynicotinic acid, nicotinate, L-aspartic acid and beta-nicotinamide mononucleotide showed an increased or insignificant change in the roots and/or leaves of foliar-exposed to NPs or ions under Cd stress (Fig. 9). These results indicated that NPs and ions modulate nicotinate and nicotinamide metabolism responsible for plant tolerance to Cd stress. The details of the regulatory mechanisms are worth further elucidation.

Furthermore, other vital metabolites, including phytohormones, phenols, carbohydrates, were also significantly affected by foliar spraying with NPs (Figs. 7, 8, Additional file 2: Table S1 and Additional file 3: Table S2, Additional file 4: Table S3 and Additional file 5: Table S4). Phenols and phenylpropanoids facilitate heavy metal fixation on the surface of phenolic or carboxylic groups, thus protecting plants against heavy metal stress [49, 72]. The metabolic pathways of these metabolites are shared with many intermediates and form complex regulatory networks [56, 64]. These findings demonstrate that more Cd-induced metabolites recovered to normal levels responsible for the performance of NPs under Cd stress, thus playing critical roles in balancing plant growth and tolerance under abiotic stress.

## Conclusion

Our study investigated the effects of Fe_3_O_4_ or ZnO NPs on plant growth and Cd responses in tobacco seedlings. Foliar exposure to Fe_3_O_4_ or ZnO NPs showed great potential in alleviating plant growth under Cd stress. Fe_3_O_4_ or ZnO NPs reprogrammed critical metabolic pathways, including alkaloids, amino acids, flavonoids, and the DAMs involved in these pathways were significantly correlated with plant growth. Notably, both Fe_3_O_4_ and ZnO NPs recovered more metabolites to normal levels under Cd stress than ions. This study will enable us to understand how Fe_3_O_4_ or ZnO NPs reprogram metabolome profiling and provide novel insights into using NPs for improving tobacco growth in Cd-contaminated soil. Further study on the underlying molecular mechanism of the foliar NPs treatment is needed in tobacco and other crops, which will provide compelling evidence for using NPs to improve crop growth and quality.

## Materials and methods

### Characterization of Fe_3_O_4_ and ZnO nanoparticles and stock preparation

Fe_3_O_4_ NPs (purity 99.9%, size 20 nm) and ZnO NPs (purity 99.9%, size 30 ± 10 nm) were purchased from Meilun Biotechnology Co., Ltd. and Macklin Biotechnological Co., Ltd., respectively. Detection was performed using transmission electron microscopy (Talos F200X, Thermo Scientific, USA). The hydrodynamic particle size and zeta potentials were determined at 50 mg·L^−1^ Fe_3_O_4_ or ZnO NPs using a dynamic light scattering (LDS) apparatus (JEM-2100, Zetasizer Nano ZS90 zeta, Malvern, England). Fe_3_O_4_ and ZnO NPs stock solutions were prepared according to previous descriptions [21, 30]. The release of iron and zinc at a series of Fe_3_O_4_ and ZnO NPs concentrations was determined as described previously [29, 31]. The ion content was detected by ICP-AES (iCAP6300, Thermo Fisher Scientific, USA).

### Seed germination and plant cultivation

The tobacco cultivar ‘Yunyan 87’ was planted at the Yuxi Breeding Base of Yunnan Academy of Tobacco Agricultural Sciences, Yunnan, China. Mature tobacco seeds were air-dried and stored at − 20 °C before use. After being disinfected with 20% (v/v) bleach for 10 min, the seeds were washed with sterile water five times and germinated on Petri dishes with two layers of filter papers soaked in ddH_2_O in a growth chamber at 26 °C under 16 h light photoperiod for 7 days. Then, 12 consistent-looking seedlings were transplanted to each pot, and the pots were covered with a plastic film that could prevent NPs from entering the solution. The seedlings were fostered in one-fourth (1/4) strength Hoagland solutions for 3 weeks. The solutions replacement was performed every 5 days.

### Foliar exposure of Fe_3_O_4_ and ZnO nanoparticles

Four-week-old ‘Yunyan 87’ seedlings with consistent growth were used for analysis. Spray bottles were used to perform foliar application of 100 mL freshly prepared 50 mg·L^−1^ Fe_3_O_4_, ZnO NPs, FeSO_4_ or ZnSO_4_ with ddH_2_O every other 3 days for five times, and 1/4 strength Hoagland solutions were added with or without 5 μM CdCl_2_, which were specified as follows: CK, control; Cd, 5 μM Cd; FeNP, 50 mg·L^−1^ Fe_3_O_4_ NPs; FeNP_Cd, 50 mg·L^−1^ Fe_3_O_4_ NPs + Cd; Fe, 50 mg·L^−1^ FeSO_4_; Fe_Cd, 50 mg·L^−1^ FeSO_4_ + Cd; ZnNP, 50 mg·L^−1^ ZnO NPs; ZnNP_Cd, 50 mg·L^−1^ ZnO NPs + Cd; Zn, 50 mg·L^−1^ ZnSO_4_; and Zn_Cd, 50 mg·L^−1^ ZnSO_4_ + Cd. Three pots, including 36 seedlings per treatment, were carried out, and about 0.695 mg NPs or ions were exposed to each plant. The solutions were renewed every 5 days, and the seedlings for additional growth for 21 days were harvested.

### Determination of plant growth parameters

After being treated for the indicated time, the plant growth parameters, including plant height, shoot fresh weight (FW), root length and FW, were characterized for each treatment/seedling. First, the plant height was measured from the junction to the shoot apex, and root length was measured to the most extended root tip. Then, the seedlings for each treatment were harvested and weighed to determine the shoot and root FW.

### Mineral element determination

The mineral elements were determined as described previously [32]. Briefly, the surface ions were chelated using 1 mM EDTA solutions for 30 min. After rinsing with ddH_2_O 5 times, the samples were fixed at 105 °C for one hour and oven-dried till steady weights at 70 °C. Approximately 0.25 mg of fine powders were used for mineral element detection. Subsequently, 10 ml HNO_3_ and 2 ml HClO_4_ were successively added and wet-washed overnight, followed by boiling for 2 h at 185 °C. Then, 4 mL of diluted HCl (v/v, HCl: ddH_2_O = 1:3) was added to the residual solution and brought to 25 mL. The mineral elements, including cadmium (Cd), potassium (K), iron (Fe), manganese (Mn), calcium (Ca), copper (Cu), magnesium (Mg), and zinc (Zn), were quantified by ICP-AES.

### Metabolomics profiling analysis

A total of 60 arrays for the roots and leaves were used for widely targeted metabolomics analyses as described previously [73, 74] and performed using a UHPLC system with a Phenomenex Kinetex column coupled to a Triple TOF 6600 instrument (QTOF, AB Sciex) by Biotree Biomedical Technology Co., Ltd. (Shanghai, China). The detailed procedures and statistical analysis were shown in Additional file 9: Supporting Information Text S1. Three replicates for each treatment were determined.

### Statistical analysis

Each experiment was performed using three independent biological repetitions. The results are the means ± standard error (SE). We performed Student’s *t*-test (IBM SPSS Statistics 20.0) to determine the significant difference between the treatments and the control, and asterisks show significant differences at *P* < 0.05. For multiple group comparisons, one-way ANOVA followed Tukey’s test was performed, and the different lowercase letters indicated significant differences at *P* < 0.05.

## Supplementary Information

**Additional** **file** **1:** **Figure** **S1.** Fe_3_O_4_ and ZnO NPs via transmission electron microscopy and dynamic light scattering. Transmission electron microscopy (TEM) imaging of Fe_3_O_4_ NPs (**a**) and ZnO NPs (**d**), bars=100 nm. Dynamic light scattering (DLS) measurements of Fe_3_O_4_ NPs (**b**) and ZnO NPs (**e**) particle size distribution by intensity. DLS measurements of zeta potential for Fe_3_O_4_ NPs (**c**) and ZnO NPs (**f**) dispersed in ddH_2_O. The different lines represent replicate measurements. The results shown are means ± SE (n=3). **Figure** **S2.** Fe and Zn contents were released from different concentrations of Fe_3_O_4_ or ZnO NP solutions, respectively. The Fe content in the Fe_3_O_4_ NP solutions (**a**) and Zn content in the ZnO NP solutions (**b**). **Figure** **S3.** OPLS-DA loading plot of metabolites in the roots and leaves of Cd-treated tobacco seedlings. CK, control; Cd, 5 μM Cd. **Figure** **S4.** OPLS-DA loading plot of metabolites in the roots among different treatments. CK, control; Cd, 5 μM Cd; FeNP, 50 mg·L^-1^ Fe_3_O_4_ NPs; FeNP_Cd, 50 mg·L^-1^ Fe_3_O_4_ NPs+Cd; Fe, 50 mg·L^-1^ FeSO_4_; Fe_Cd, 50 mg·L^-1^ FeSO_4_+Cd; ZnNP, 50 mg·L^-1^ ZnO NPs; ZnNP_Cd, 50 mg·L^-1^ ZnO NPs+Cd; 50 mg·L^-1^ ZnSO_4_; and Zn_Cd, 50 mg·L^-1^ ZnSO_4_+Cd. **Figure** **S5.** OPLS-DA loading plot of metabolites in the leaves among different treatments. CK, control; Cd, 5 μM Cd; FeNP, 50 mg·L^-1^ Fe_3_O_4_ NPs; FeNP_Cd, 50 mg·L^-1^ Fe_3_O_4_ NPs+Cd; Fe, 50 mg·L^-1^ FeSO_4_; Fe_Cd, 50 mg·L^-1^ FeSO_4_+Cd; ZnNP, 50 mg·L^-1^ ZnO NPs; ZnNP_Cd, 50 mg·L^-1^ ZnO NPs+Cd; 50 mg·L^-1^ ZnSO_4_; and Zn_Cd, 50 mg·L^-1^ ZnSO_4_+Cd. **Figure** **S6.** Venn analysis of differentially accumulated metabolites in the roots and leaves of tobacco seedlings exposed to Cd stress. Cd, 5 μM Cd. Up, upregulated metabolites; Down, downregulated metabolites (Cd/control). **Figure** **S7.** Venn analysis of differentially accumulated metabolites in the roots and leaves of tobacco seedlings exposed to Fe_3_O_4_ NPs or FeSO_4_. Four-week-old tobacco seedlings were transferred to 1/4 strength fresh Hoagland solutions, and their foliage was exposed to 50 mg L^-1^ Fe_3_O_4_ NPs (**a**) or FeSO_4_ (**b**) for 21 days. FeNP, 50 mg·L^-1^ Fe_3_O_4_ NPs; Fe, 50 mg·L^-1^ FeSO_4_. Up, upregulated metabolites; Down, downregulated metabolites. **Figure** **S8.** Venn analysis of differentially accumulated metabolites in the roots and leaves of Cd-treated tobacco seedlings exposed to Fe_3_O_4_ NPs or FeSO_4_. Four-week-old tobacco seedlings were transferred to 1/4 strength fresh Hoagland solutions supplemented with 5 μM CdCl_2_, and their foliage was exposed to 50 mg L^-1^ Fe_3_O_4_ NPs (**a**) or FeSO_4_ (**b**) for 21 days. Cd, 5 μM Cd; FeNP_Cd, 50 mg·L^-1^ Fe_3_O_4_ NPs+Cd; Fe_Cd, 50 mg·L^-1^ FeSO_4_+Cd. Up, upregulated metabolites; Down, downregulated metabolites. **Figure** **S9.** Venn analysis of differentially accumulated metabolites in the roots and leaves of tobacco seedlings exposed to ZnO NPs or ZnSO_4_. Four-week-old seedlings were transferred to 1/4 strength fresh Hoagland solutions, and their foliage was exposed to 50 mg·L^-1^ ZnO NPs (**a**) or ZnSO_4_ (**b**) for 21 days. ZnNP, 50 mg·L^-1^ ZnO NPs; Zn, 50 mg·L^-1^ ZnSO_4_. Up, upregulated metabolites; Down, downregulated metabolites. **Figure** **S10.** Venn analysis of differentially accumulated metabolites in the roots and leaves of Cd-treated tobacco seedlings exposed to ZnO NPs or ZnSO_4_. Four-week-old tobacco seedlings were transferred to 1/4 strength fresh Hoagland solutions supplemented with 5 μM CdCl_2_, and foliar exposed to 50 mg·L^-1^ ZnO NPs (**a**) or ZnSO_4_ (**b**) for 21 days. Cd, 5 μM Cd; ZnNP_Cd, 50 mg·L^-1^ ZnO NPs+Cd; Zn_Cd, 50 mg·L^-1^ ZnSO_4_+Cd. Up, upregulated metabolites; Down, downregulated metabolites. **Figure** **S11.** Venn analysis of differentially accumulated metabolites in the roots and/or leaves of tobacco seedlings exposed to Fe_3_O_4_ or ZnO NPs. FeNP, 50 mg·L^-1^ Fe_3_O_4_ NPs; ZnNP, 50 mg·L^-1^ ZnO NPs. Up, upregulated metabolites; Down, downregulated metabolites. **Figure** **S12.** Venn analysis of differentially accumulated metabolites in the roots and/or leaves of Cd-treated tobacco seedlings exposed to Fe_3_O_4_ or ZnO NPs. Cd, 5μM Cd; FeNP_Cd, 50 mg·L^-1^ Fe_3_O_4_ NPs+Cd; ZnNP_Cd, 50 mg·L^-1^ ZnO NPs+Cd. Up, upregulated metabolites; Down, downregulated metabolites. **Figure** **S13.** Venn analysis of differentially accumulated metabolites in the roots and/or leaves of tobacco seedlings exposed to FeSO_4_ or ZnSO_4_. Fe, 50 mg·L^-1^ FeSO_4_; Zn, 50 mg·L^-1^ ZnSO_4_. Up, upregulated metabolites; Down, downregulated metabolites. **Figure** **S14.** Venn analysis of differentially accumulated metabolites in the roots and/or leaves of Cd-treated tobacco seedlings exposed to FeSO_4_ or ZnSO_4_. Cd, 5μM Cd; Fe_Cd, 50 mg·L^-1^ FeSO_4_+Cd; Zn_Cd, 50 mg·L^-1^ ZnSO_4_+Cd. Up, upregulated metabolites; Down, downregulated metabolites. **Figure** **S15.** Venn analysis of differentially accumulated metabolites in the roots and/or leaves of tobacco seedlings exposed to Fe_3_O_4_ NPs or FeSO_4_. FeNP, 50 mg·L^-1^ Fe_3_O_4_ NPs; Fe, 50 mg·L^-1^ FeSO_4_. Up, upregulated metabolites; Down, downregulated metabolites. **Figure** **S16.** Venn analysis of differentially accumulated metabolites in the roots and/or leaves of Cd-treated tobacco seedlings exposed to Fe_3_O_4_ NPs or FeSO_4_. Cd, 5μM Cd; FeNP_Cd, 50 mg·L^-1^ Fe_3_O_4_ NPs+Cd; Fe_Cd, 50 mg·L^-1^ FeSO_4_+Cd. Up, upregulated metabolites; Down, downregulated metabolites. **Figure** **S17.** Venn analysis of differentially accumulated metabolites in the roots and/or leaves of tobacco seedlings exposed to ZnO NPs or ZnSO_4_. ZnNP, 50 mg·L^-1^ ZnO NPs; Zn, 50 mg·L^-1^ ZnSO_4_. Up, upregulated metabolites; Down, downregulated metabolites. **Figure** **S18.** Venn analysis of differentially accumulated metabolites in the roots and/or leaves of Cd-treated tobacco seedlings exposed to ZnO NPs or ZnSO_4_. Cd, 5μM Cd; ZnNP_Cd, 50 mg·L^-1^ ZnO NPs+Cd; Zn_Cd, 50 mg·L^-1^ ZnSO_4_+Cd. Up, upregulated metabolites; Down, downregulated metabolites.

**Additional** **file** **2:** **Table** **S1.** The differentially accumulated metabolites in the roots of ‘Yunyan 87’ ( *Nicotiana* *tabacum*) seedlings.

**Additional** **file** **3:** **Table** **S2.** The differentially accumulated metabolites in the leaves of ‘Yunyan 87’ ( *Nicotiana* *tabacum*) seedlings.

**Additional** **file** **4:** **Table** **S3.** The Cd-induced differentially accumulated metabolites in the roots of ‘Yunyan 87’ (*Nicotiana* *tabacum*) seedlings.

**Additional** **file** **5:** **Table** **S4.** The Cd-induced differentially accumulated metabolites in the leaves of ‘Yunyan 87’ (*Nicotiana* *tabacum*) seedlings.

**Additional** **file** **6:** **Table** **S5.** Correlation analysis of differentially accumulated metabolites in the roots and plant growth parameters (Root length, Root-FW).

**Additional** **file** **7:** **Table** **S6.** Correlation analysis of differentially accumulated metabolites in the leaves and plant growth parameters (Plant height, Shoot-FW).

**Additional** **file** **8:** **Table** **S7.** Significant correlations between metabolites and growth parameters in both root and leaves.

**Additional** **file** **9:** **Text** **S1.** Supporting information.

## Data Availability

The datasets used and/or analyzed in this paper are available from the corresponding authors upon reasonable request. In addition, the original metabolome datasets generated in the current study are available in Additional file 2: Table S1, Additional file 3: Table S2, Additional file 4: Table S3, Additional file 5: Table S4, Additional file 6: Table S5, Additional file 7: Table S6, Additional file 8: Table S7.

## References

[1] Zhao F-J, Tang Z, Song J-J, Huang X-Y, Wang P (2021). Toxic metals and metalloids: uptake, transport, detoxification, phytoremediation and crop improvement for safer food. Mol Plant.

[2] Lin Q, Dai W, Chen J-Q, Jin Y, Yang Y, Wang Y-Y, Zhang B-F, Fan J-M, Lou L-P, Shen Z-G, Shen C-F, Mao J-D (2021). Airborne lead: a vital factor influencing rice lead accumulation in China. J Hazard Mater.

[3] Wang P, Chen H, Zhao F-J (2019). Cadmium contamination in agricultural soils of China and the impact on food safety. Environ Pollut.

[4] Yang Z, Yang F, Liu J, Wu H, Yang H, Shi Y, Liu J, Zhang Y, Luo Y, Chen K (2022). Heavy metal transporters: functional mechanisms, regulation, and application in phytoremediation. Sci Total Environ.

[5] Cheng S (2003). Effects of heavy metals on plants and resistance mechanisms. Environ Sci Pollut.

[6] Dalcorso G, Farinati S, Furini A (2010). Regulatory networks of cadmium stress in plants. Plant Signal Behav.

[7] Cobbett C, Goldsbrough P (2002). Phytochelatins and metallothioneins: roles in heavy metal detoxification and homeostasis. Annu Rev Plant Biol.

[8] Cunningham F, Goh N, Demirer GS, Matos JL, Landry MP (2018). Nanoparticle-mediated delivery towards advancing plant genetic engineering. Trends Biotechnol.

[9] Yan Y, Zhu X, Yu Y, Li C, Zhang Z, Wang F. Nanotechnology strategies for plant genetic engineering. Adv Mater. 2022;34:2106945 10.1002/adma.202106945 34699644

[10] Salata V (2004). Applications of nanoparticles in biology and medicine. J Nanobiotechnol.

[11] Liu J, Li G, Chen L, Gu J, Wu H, Li Z (2021). Cerium oxide nanoparticles improve cotton salt tolerance by enabling better ability to maintain cytosolic K^+^/Na^+^ ratio. J Nanobiotechnol.

[12] Wu H, Li Z (2021). Recent advances in nano-enabled agriculture for improving plant performance. Crop J.

[13] Verma SK, Das AK, Patel MK, Shah A, Kumar V, Gantait S (2018). Engineered nanomaterials for plant growth and development: a perspective analysis. Sci Total Environ.

[14] Zhao L, Lu L, Wang A, Zhang H, Huang M, Wu H, Xing B, Wang Z, Ji R (2020). Nanobiotechnology in agriculture: use of nanomaterials to promote plant growth and stress tolerance. J Agric Food Chem.

[15] Zhou P, Adeel M, Shakoor N, Guo M, Hao Y, Azeem I, Li M, Liu M, Rui Y (2020). Application of nanoparticles alleviates heavy metals stress and promotes plant growth: an overview. Nanomaterials.

[16] Liu J, Zhao Z, Jiang G (2008). Coating Fe_3_O_4_ magnetic nanoparticles with humic acid for high efficient removal of heavy metals in water. Environ Sci Technol.

[17] Hua M, Zhang S, Pan B, Zhang W, Lv L, Zhang Q (2011). Heavy metal removal from water/wastewater by nanosized metal oxides: a review. J Hazard Mater.

[18] Li J, Hu J, Ma C, Wang Y, Wu C, Huang J, Xing B (2016). Uptake, translocation and physiological effects of magnetic iron oxide (γ-Fe_2_O_3_) nanoparticles in corn (*Zea*
*mays* L.). Chemosphere.

[19] Ghafariyan MH, Malakouti MJ, Dadpour MR, Stroeve P, Mahmoudi M (2013). Effects of magnetite nanoparticles on soybean chlorophyll. Environ Sci Technol.

[20] Alidoust D, Isoda A (2013). Effect of γFe_2_O_3_ nanoparticles on photosynthetic characteristic of soybean (Glycine max (L.) Merr.): foliar spray versus soil amendment. Acta Physiol Plant.

[21] Wang H, Kou X, Pei Z, Xiao JQ, Shan X, Xing B (2011). Physiological effects of magnetite (Fe_3_O_4_) nanoparticles on perennial ryegrass (*Lolium*
*perenne* L.) and pumpkin (*Cucurbita*
*mixta*) plants. Nanotoxicology.

[22] Hussain A, Ali S, Zia-ur-Rehman M, Qayyum M, Wang H, Rinklebe J (2019). Responses of wheat (*Triticum*
*aestivum*) plants grown in a Cd contaminated soil to the application of iron oxide nanoparticles. Ecotox Environ Safe.

[23] Konnte A, He X, Ma Y, Yang J, Alugongo GM, Rui Y, Zhang Z (2018). Alleviation of cadmium-induced changes on growth, antioxidative enzyme activities and lipid peroxidation in crop seedings by magnetic (Fe_3_O_4_) nanoparticles. Fresenius Environ Bull.

[24] Konate A, He X, Ma Y, Alugongo G, Yukui R (2017). Magnetic (Fe_3_O_4_) nanoparticles reduce heavy metals uptake and mitigate their toxicity in wheat seedling. Sustainability.

[25] Rao S, Shekhawat GS (2013). Toxicity of ZnO engineered nanoparticles and evaluation of their effect on growth, metabolism and tissue specific accumulation in *Brassica*
*juncea*. J Environ Chem.

[26] Lv J, Zhang S, Luo L, Zhang J, Yang K, Christie P (2015). Accumulation, speciation and uptake pathway of ZnO nanoparticles in maize. Environ Sci Nano.

[27] Lin D, Xing B (2008). Root uptake and phytotoxicity of ZnO nanoparticles. Environ Sci Technol.

[28] Landa P, Prerostova S, Petrova S, Knirsch V, Vanková R, Vanek T (2015). The transcriptomic response of Arabidopsis thaliana to zinc oxide: a comparison of the impact of nanoparticle, bulk, and ionic zinc. Environ Sci Technol.

[29] Nair PMG, Chung IM (2017). Regulation of morphological, molecular and nutrient status in *Arabidopsis*
*thaliana* seedlings in response to ZnO nanoparticles and Zn ion exposure. Sci Total Environ.

[30] Wan J, Wang R, Wang R, Ju Q, Wang Y, Xu J (2019). Comparative physiological and transcriptomic analyses reveal the toxic effects of ZnO nanoparticles on plant growth. Environ Sci Technol.

[31] Sun L, Wang Y, Wang R, Wang R, Zhang P, Ju Q, Xu J (2020). Physiological, transcriptomic, and metabolomic analyses reveal zinc oxide nanoparticles modulate plant growth in tomato. Environ Sci Nano.

[32] Wan J, Wang R, Bai H, Wang Y, Xu J (2020). Comparative physiological and metabolomics analysis reveals that single-walled carbon nanohorns and ZnO nanoparticles affect salt tolerance in *Sophora*
*alopecuroides*. Environ Sci Nano.

[33] Rizwan M, Ali S, Zia ur Rehman M, Adrees M, Arshad M, Qayyum MF, Ali L, Hussain A, Chatha SAS, Imran M (2019). Alleviation of cadmium accumulation in maize (*Zea*
*mays* L.) by foliar spray of zinc oxide nanoparticles and biochar to contaminated soil. Environ Pollut.

[34] Ma X, Sharifan H, Dou F, Sun W (2020). Simultaneous reduction of arsenic (As) and cadmium (Cd) accumulation in rice by zinc oxide nanoparticles. Chem Eng J.

[35] Li Y, Liang L, Wu L, Ashraf U, Ma L, Tang X, Pan S, Tian H, Mo Z (2021). ZnO nanoparticle-based seed priming modulates early growth and enhances physio-biochemical and metabolic profiles of fragrant rice against cadmium toxicity. J Nanobiotechnol.

[36] Jiao F, Zhao L, Wu X, Song Z, Li Y (2020). Metabolome and transcriptome analyses of the molecular mechanisms of flower color mutation in tobacco. BMC Genom.

[37] Huang Z, Zhao T, Wang N, Zheng S (2016). Ectopic expression of Lc differentially regulated anthocyanin biosynthesis in the floral parts of tobacco (*Nicotiana*
*tobacum* L.) plants. Bot Stud.

[38] Zhang B, Shang S, Jabeen Z, Zhang GP (2014). Involvement of ethylene in alleviation of Cd toxicity by NaCl in tobacco plants. Ecotox Environ Safe.

[39] Lugon-Moulin N, Calvino-Martin F, Krauss M, Ramey P, Rossi L (2006). Cadmium concentration in tobacco (*Nicotiana*
*tabacum* L.) from different countries and its relationship with other elements. Chemosphere.

[40] Gao Y-L, Yao X-F, Song X-F, Li W-Z, Song Z-B, Wang B-W, Wu Y-P, Shi J-L, Liu G-S, Li Y-P, Liu C-M (2019). An efficient TILLING platform for cultivated tobacco. Integr Plant Biol.

[41] Li C, Feng P, Li D, Brestic M, Liu Y, Yang X (2021). Genetic engineering of glycinebetaine synthesis enhances cadmium tolerance in BADH-transgenic tobacco plants via reducing cadmium uptake and alleviating cadmium stress damage. Environ Exp Bot.

[42] Zhang Y, Chao J, Li X, Zhang C, Khan R, Du S, Xu N, Song L, Liu H, Shi Y (2021). Comparative transcriptome combined with biochemical and physiological analyses provide new insights toward cadmium accumulation with two contrasting *Nicotiana* species. Physiol Plant.

[43] Palusińska M, Barabasz A, Kozak K, Papierniak A, Maślińska K, Antosiewicz D (2020). Zn/Cd status-dependent accumulation of Zn and Cd in root parts in tobacco is accompanied by specific expression of *ZIP* genes. BMC Plant Biol.

[44] Hao Y, Yuan W, Ma C, White J, Zhang Z, Adeel M, Zhou T, Rui Y, Xing B (2018). Engineered nanomaterials suppress Turnip mosaic virus infection in tobacco (*Nicotiana*
*benthamiana* ). Environ Sci Nano.

[45] Alkhatib R, Alkhatib B, Abdo N (2021). Effect of Fe_3_O_4_ nanoparticles on seed germination in tobacco. Environ Sci Pollut Res.

[46] Matsui A, Yin Y, Yamanaka K, Iwasaki M, Ashihara H (2007). Metabolic fate of nicotinamide in higher plants. Physiol Plant.

[47] Kanehisa M, Sato Y, Kawashima M (2021). KEGG mapping tools for uncovering hidden features in biological data. Protein Sci.

[48] Fan X, Zhou X, Chen H, Tang M, Xie X (2021). Cross-talks between macro- and micronutrient uptake and signaling in plants. Front Plant Sci.

[49] Wang J, Chen X, Chu S, You Y, Chi Y, Wang R, Yang X, Hayat K, Zhang D, Zhou P (2022). Comparative cytology combined with transcriptomic and metabolomic analyses of *Solanum*
*nigrum* L. in response to Cd toxicity. J Hazard Mater.

[50] Miller A-F (2011). Superoxide dismutase: ancient enzymes and new insights. FEBS Lett.

[51] Chen Z, Shijia L, Li J, Chen T, Gu Q, Yang T, Zhang Z (2021). Theanine improves salt stress tolerance via modulating redox homeostasis in tea plants (*Camellia*
*sinensis* L.). Front Plant Sci.

[52] Wang C, Zhao Y, Gu P, Zou F, Meng L, Song W, Yang Y, Wang S, Yali Z (2017). Auxin is involved in lateral root formation induced by drought stress in tobacco seedlings. J Plant Growth Regul.

[53] Agami R, Farag G (2013). Exogenous treatment with indole-3-acetic acid and salicylic acid alleviates cadmium toxicity in wheat seedlings. Ecotox Environ Safe.

[54] Li Y, Zhang Y, Shi D, Liu X, Qin J, Ge Q, Xu L, Pan X, Li W, Zhu Y, Xu J (2013). Spatial-temporal analysis of zinc homeostasis reveals the response mechanisms to acute zinc deficiency in *Sorghum*
*bicolor*. New Phytol.

[55] Pietrowska-Borek M, Wojdyła A, Dobrogojski J, Młynarska-Cieślak A, Baranowski M, Dąbrowski J, Kowalska J, Jemielity J, Borek S, Pedreño M, Guranowski A (2019). Purine and pyrimidine dinucleoside polyphosphates differentially affect the phenylpropanoid pathway in *Vitis*
*vinifera* L. cv. Monastrell suspension cultured cells. Plant Physiol Biochem.

[56] Kliebenstein D, Osbourn A (2012). Making new molecules—evolution of pathways for novel metabolites in plants. Curr Opin Plant Biol.

[57] Pichersky E, Gang D (2000). Genetics and biochemistry of secondary metabolites in plants: an evolutionary perspective. Trends Plant Sci.

[58] Yang Q, Zhao D, Liu Q (2020). Connections between amino acid metabolisms in plants: lysine as an example. Front Plant Sci.

[59] Zheng J, Zhao L, Wu C, Shen B, Zhu A (2015). Exogenous proline reduces NaCl-induced damage by mediating ionic and osmotic adjustment and enhancing antioxidant defense in *Eurya*
*emarginata*. Acta Physiol Plant.

[60] Wang G, Kong J, Cui D, Zhao H, Niu Y, Xu M, Jiang G, Zhao Y, Wang W (2018). Resistance against *Ralstonia*
*solanacearum* in tomato depends on methionine cycle and γ-aminobutyric acid metabolic pathways. Plant J.

[61] Yang L, Wen K-S, Ruan X, Zhao Y-X, Wei F, Wang Q (2018). Response of plant secondary metabolites to environmental factors. Molecules.

[62] Yeshi K, Ritmejerytė E, Wangchuk P, Yeshi K, Crayn D, Ritmejerytė E, Wangchuk P (2022). Secondary metabolites produced in response to abiotic stresses has potential application in pharmaceutical product development. Molecules.

[63] Siemens D, Garner S, Mitchell-Olds T, Callaway R (2002). Cost of defense in the context of plant competition: *Brassica*
*rapa* may grow and defend. Ecology.

[64] Neilson E, Goodger J, Woodrow I, Møller B (2013). Plant chemical defense: at what cost?. Trends Plant Sci.

[65] Tan P, Zeng C, Wan C, Liu Z, Dong X, Jiqing P, Lin H, Li M, Liu Z, Yan M (2021). Metabolic profiles of *Brassica*
*juncea* roots in response to cadmium stress. Metabolites.

[66] Srivastava N, Srivastava AK (2010). Influence of some heavy metals on growth, alkaloid content and composition in *Catharanthus*
*roseus* L.. Indian J Pharm Sci.

[67] Wen W, Alseekh S, Fernie A (2020). Conservation and diversification of flavonoid metabolism in the plant kingdom. Curr Opin Plant Biol.

[68] Brown D, Rashotte A, Murphy A, Normanly J, Tague B, Peer W, Taiz L, Muday G (2001). Flavonoids act as negative regulators of auxin transport in vivo in *Arabidopsis*. Plant Physiol.

[69] Wan J, Zhang P, Wang R, Sun L, Wang W, Zhou H, Xu J (2018). UV-B radiation induces root bending through the flavonoid-mediated auxin pathway in *Arabidopsis*. Front Plant Sci.

[70] Dutilleul C, Garmier M, Noctor G, Mathieu C, Chétrit P, Foyer C, de Paepe R (2003). Leaf mitochondria modulate whole cell redox homeostasis, set antioxidant capacity, and determine stress resistance through altered signaling and diurnal regulation. Plant Cell.

[71] Dutilleul C, Driscoll S, Cornic G, de Paepe R, Foyer C, Noctor G (2003). Functional mitochondrial complex I is required by tobacco leaves for optimal photosynthetic performance in photorespiratory conditions and during transients. Plant Physiol.

[72] Qiao K, Liang S, Wang F, Wang H, Hu Z, Chai T (2019). Effects of cadmium toxicity on diploid wheat (*Triticum*
*urartu*) and the molecular mechanism of the cadmium response. J Hazard Mater.

[73] Chong J, Soufan O, Li C, Caraus I, Li S, Bourque G, Wishart DS, Xia J (2018). MetaboAnalyst 4.0: towards more transparent and integrative metabolomics analysis. Nucleic Acids Res.

[74] Mamat A, Tursun K, Xu J (2021). Identification of metabolic pathways related to rough-skinned fruit formation in Korla pear. Sci Hortic.

