# Supplementary material for: Comparative physiological and metabolomic analyses reveal that Fe3O4 and ZnO nanoparticles alleviate Cd toxicity in tobacco
Source: J Nanobiotechnology. 2022 Jun 27;20:302. doi: 10.1186/s12951-022-01509-3 (PMC9235244; doi:10.1186/s12951-022-01509-3)
Supplement: Supplementary file 1 — Additional file 1: Figure S1. Fe3O4 and ZnO NPs via transmission electron microscopy and dynamic light scattering. Transmission electron microscopy (TEM) imaging of Fe3O4 NPs (a) and ZnO NPs (d), bars=100 nm. Dynamic light scattering (DLS) measurements of Fe3O4 NPs (b) and ZnO NPs (e) particle size distribution by intensity. DLS measurements of zeta potential for Fe3O4 NPs (c) and ZnO NPs (f) dispersed in ddH2O. The different lines represent replicate measurements. The results shown are means ± SE (n=3). Figure S2. Fe and Zn contents were released from different concentrations of Fe3O4 or ZnO NP solutions, respectively. The Fe content in the Fe3O4 NP solutions (a) and Zn content in the ZnO NP solutions (b). Figure S3. OPLS-DA loading plot of metabolites in the roots and leaves of Cd-treated tobacco seedlings. CK, control; Cd, 5 μM Cd. Figure S4. OPLS-DA loading plot of metabolites in the roots among different treatments. CK, control; Cd, 5 μM Cd; FeNP, 50 mg·L-1 Fe3O4 NPs; FeNP_Cd, 50 mg·L-1 Fe3O4 NPs+Cd; Fe, 50 mg·L-1 FeSO4; Fe_Cd, 50 mg·L-1 FeSO4+Cd; ZnNP, 50 mg·L-1 ZnO NPs; ZnNP_Cd, 50 mg·L-1 ZnO NPs+Cd; 50 mg·L-1 ZnSO4; and Zn_Cd, 50 mg·L-1 ZnSO4+Cd. Figure S5. OPLS-DA loading plot of metabolites in the leaves among different treatments. CK, control; Cd, 5 μM Cd; FeNP, 50 mg·L-1 Fe3O4 NPs; FeNP_Cd, 50 mg·L-1 Fe3O4 NPs+Cd; Fe, 50 mg·L-1 FeSO4; Fe_Cd, 50 mg·L-1 FeSO4+Cd; ZnNP, 50 mg·L-1 ZnO NPs; ZnNP_Cd, 50 mg·L-1 ZnO NPs+Cd; 50 mg·L-1 ZnSO4; and Zn_Cd, 50 mg·L-1 ZnSO4+Cd. Figure S6. Venn analysis of differentially accumulated metabolites in the roots and leaves of tobacco seedlings exposed to Cd stress. Cd, 5 μM Cd. Up, upregulated metabolites; Down, downregulated metabolites (Cd/control). Figure S7. Venn analysis of differentially accumulated metabolites in the roots and leaves of tobacco seedlings exposed to Fe3O4 NPs or FeSO4. Four-week-old tobacco seedlings were transferred to 1/4 strength fresh Hoagland solutions, and their foliage was exposed to 50 mg L-1 F [file 12951_2022_1509_MOESM1_ESM.docx]

**
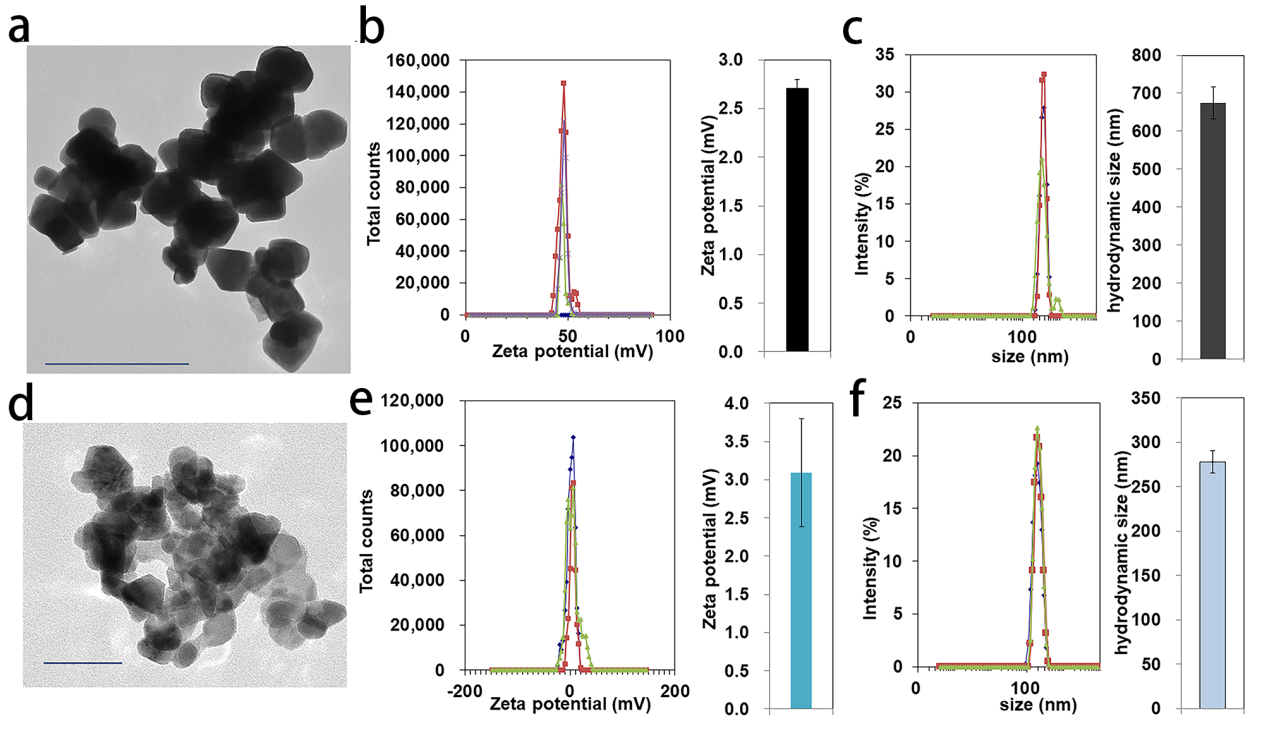
Figure S1. Fe_3_O_4_ and ZnO NPs via transmission electron microscopy and dynamic light scattering.** Transmission electron microscopy (TEM) imaging of Fe_3_O_4_ NPs (**a**) and ZnO NPs (**d**), bars=100 nm. Dynamic light scattering (DLS) measurements of Fe_3_O_4_ NPs (**b**) and ZnO NPs (**e**) particle size distribution by intensity. DLS measurements of zeta potential for Fe_3_O_4_ NPs (**c**) and ZnO NPs (**f**) dispersed in ddH_2_O. The different lines represent replicate measurements. The results shown are means ± SE (n=3).


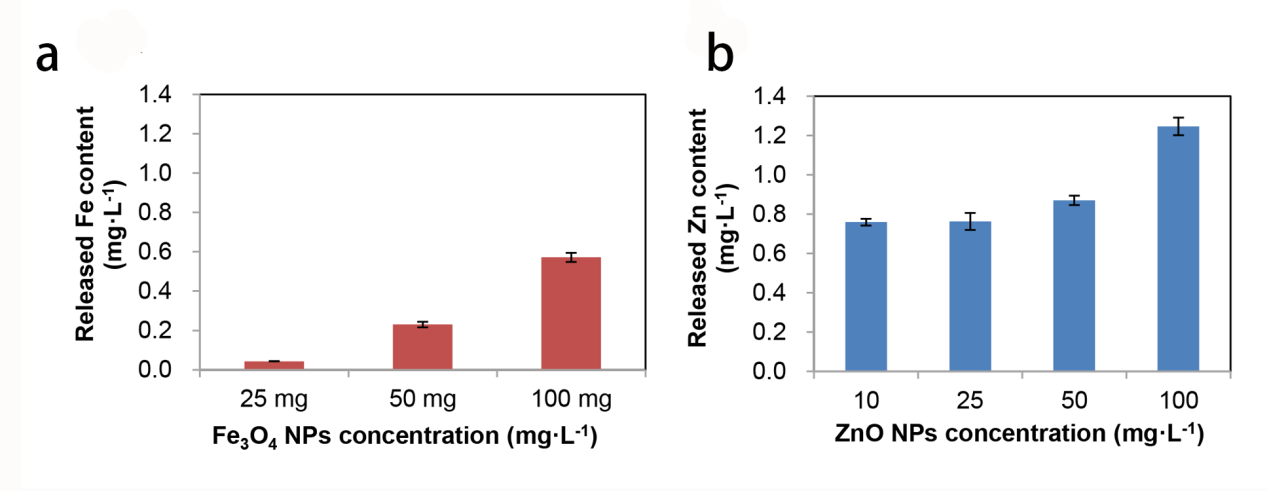
**Figure S2. Fe and Zn contents were released from different concentrations of Fe_3_O_4_ or ZnO NP solutions, respectively.** The Fe content in the Fe_3_O_4_ NP solutions (**a**) and Zn content in the ZnO NP solutions (**b**)**.**

**
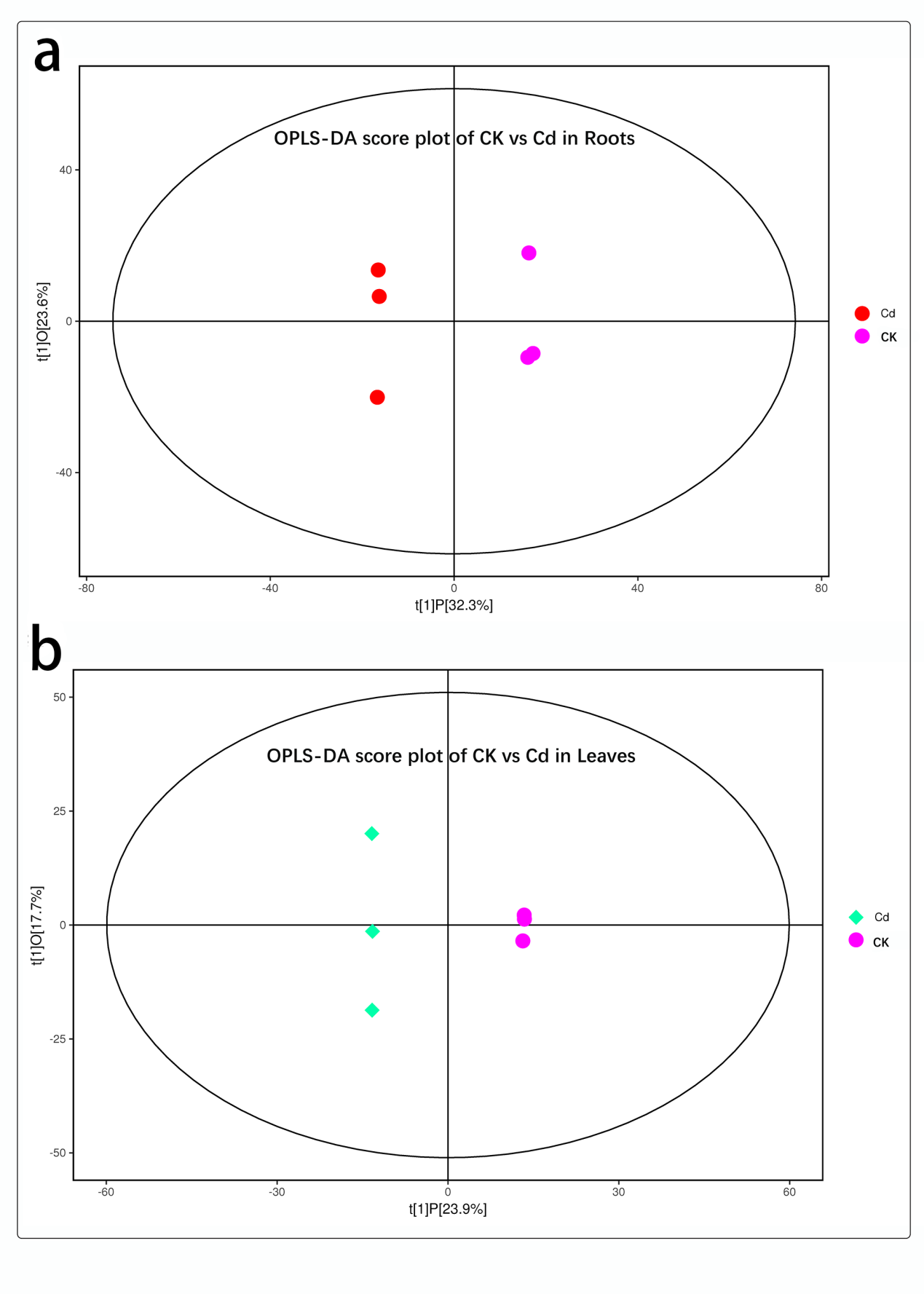
Figure S3. OPLS-DA loading plot of metabolites in the roots and leaves of Cd-treated tobacco seedlings.** CK, control; Cd, 5 μM Cd.


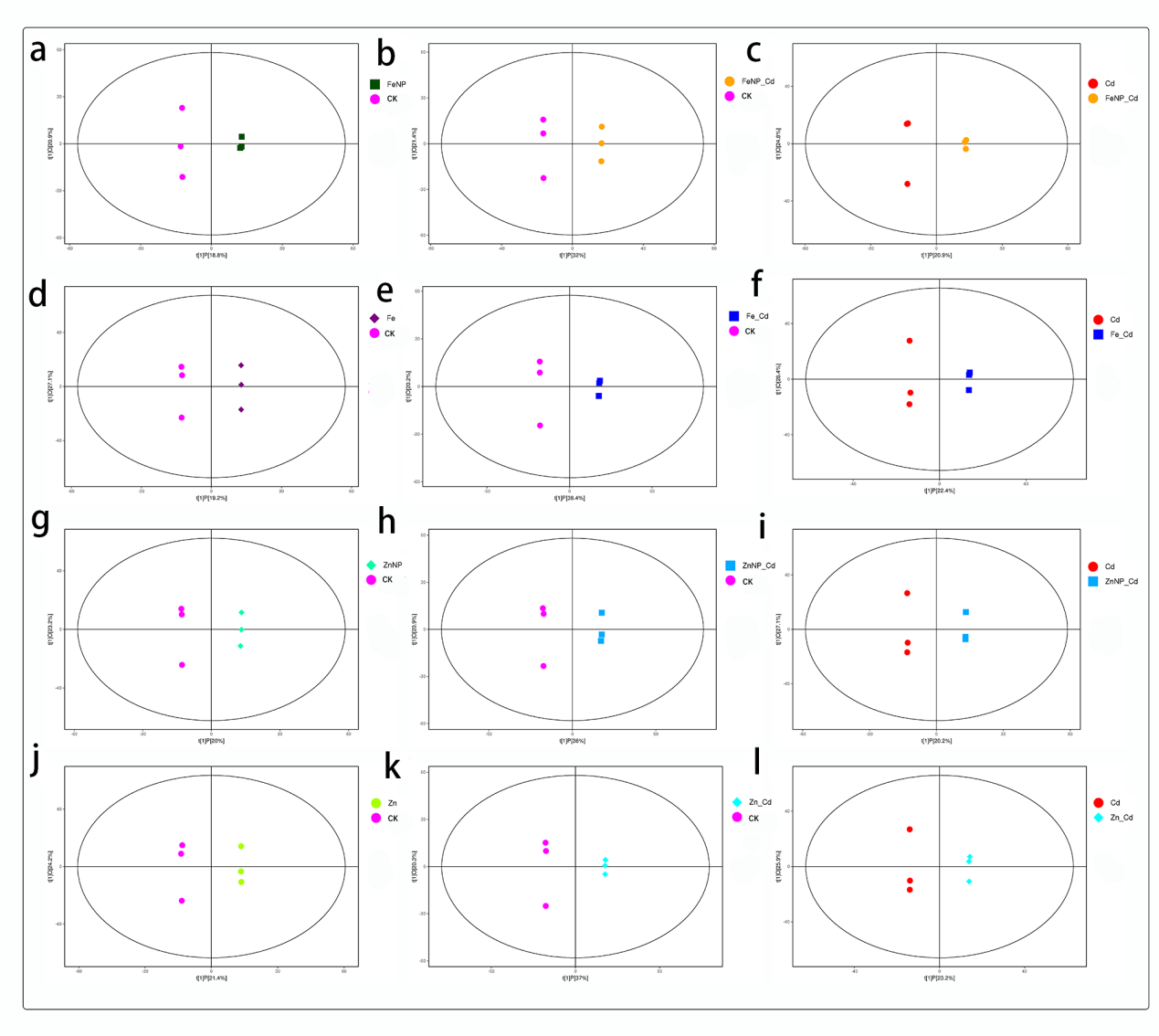
**Figure S4. OPLS-DA loading plot of metabolites in the roots among different treatments.** CK, control; Cd, 5 μM Cd; FeNP, 50 mg·L^-1^ Fe_3_O_4_ NPs; FeNP_Cd, 50 mg·L^-1^ Fe_3_O_4_ NPs+Cd; Fe, 50 mg·L^-1^ FeSO_4_; Fe_Cd, 50 mg·L^-1^ FeSO_4_+Cd; ZnNP, 50 mg·L^-1^ ZnO NPs; ZnNP_Cd, 50 mg·L^-1^ ZnO NPs+Cd; 50 mg·L^-1^ ZnSO_4_; and Zn_Cd, 50 mg·L^-1^ ZnSO_4_+Cd.

**
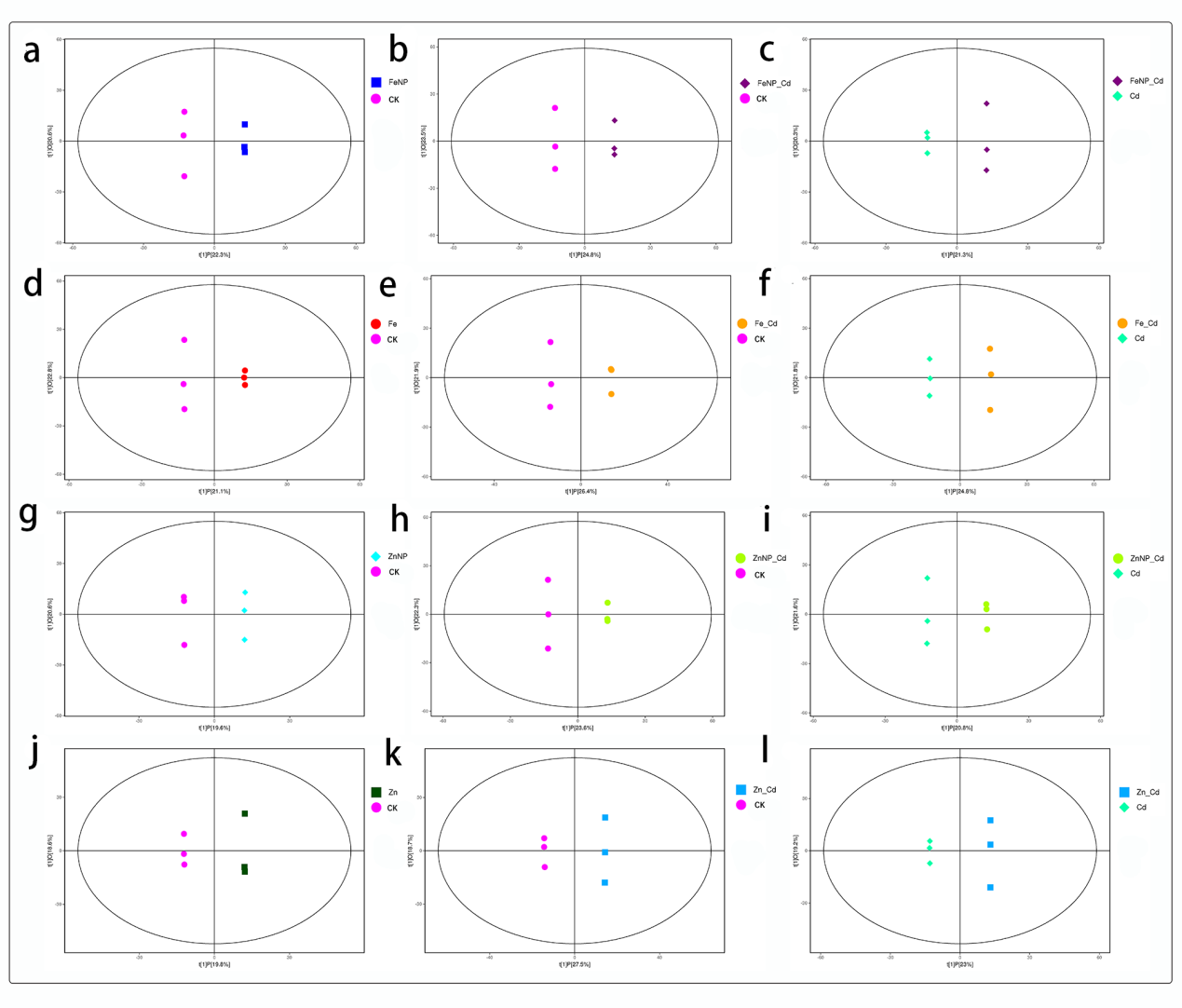
Figure S5. OPLS-DA loading plot of metabolites in the leaves among different treatments**. CK, control; Cd, 5 μM Cd; FeNP, 50 mg·L^-1^ Fe_3_O_4_ NPs; FeNP_Cd, 50 mg·L^-1^ Fe_3_O_4_ NPs+Cd; Fe, 50 mg·L^-1^ FeSO_4_; Fe_Cd, 50 mg·L^-1^ FeSO_4_+Cd; ZnNP, 50 mg·L^-1^ ZnO NPs; ZnNP_Cd, 50 mg·L^-1^ ZnO NPs+Cd; 50 mg·L^-1^ ZnSO_4_; and Zn_Cd, 50 mg·L^-1^ ZnSO_4_+Cd.

**
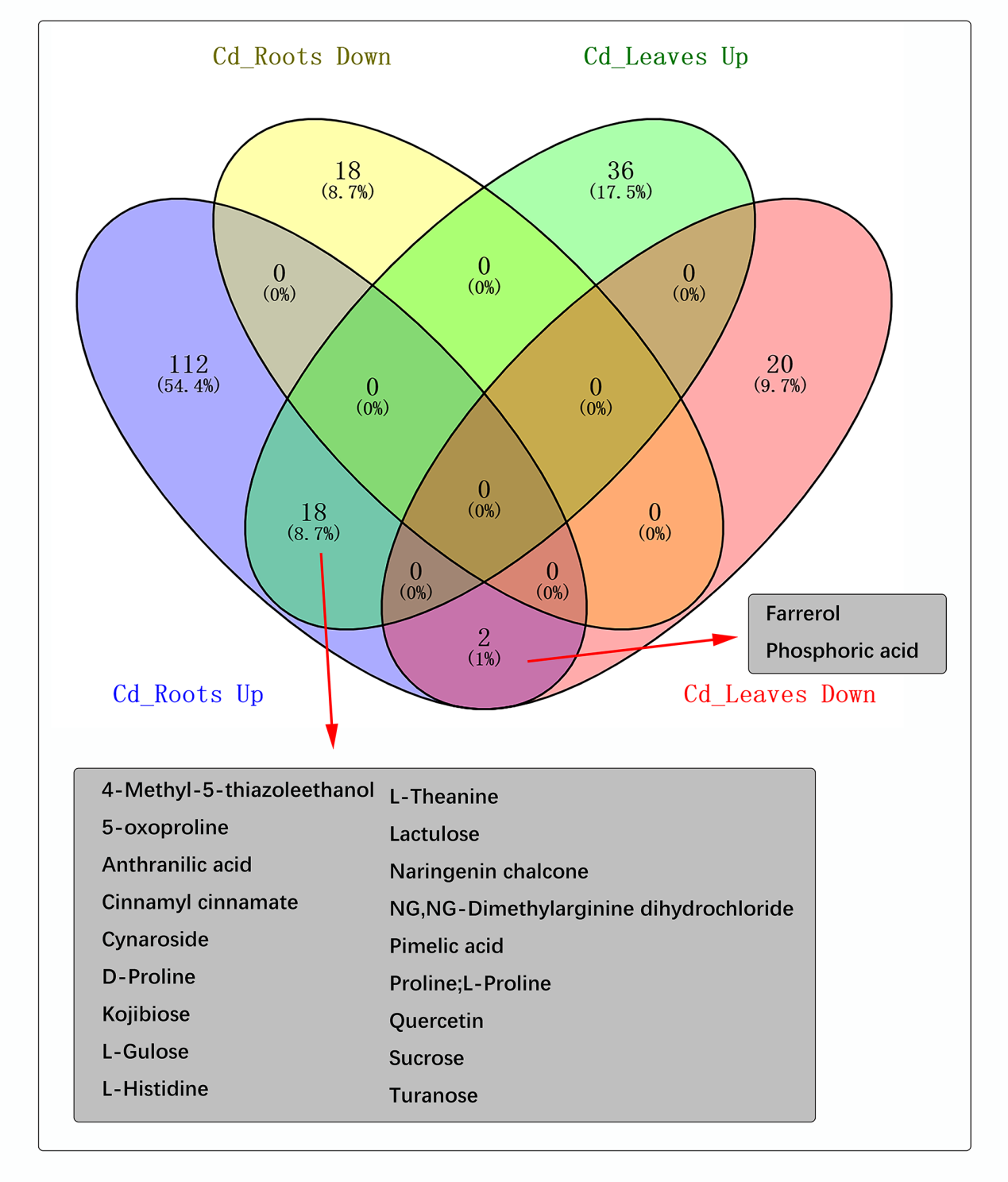
Figure S6. Venn analysis of differentially accumulated metabolites in the roots and leaves of tobacco seedlings exposed to Cd stress.** Cd, 5 μM Cd. Up, upregulated metabolites; down, downregulated metabolites (Cd/control).

**
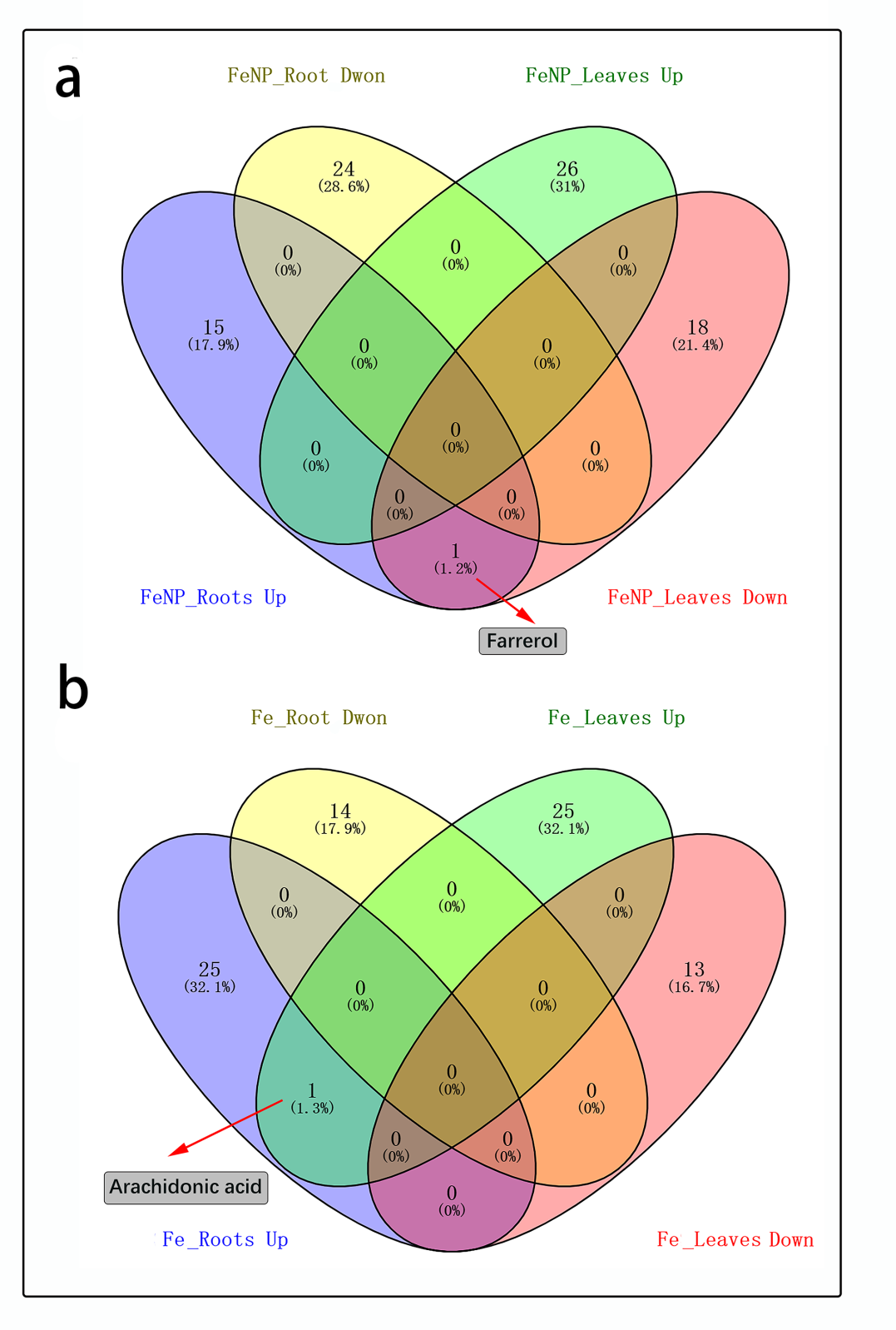
Figure S7. Venn analysis of differentially accumulated metabolites in the roots and leaves of tobacco seedlings exposed to Fe_3_O_4_ NPs or FeSO_4_.** Four-week-old tobacco seedlings were transferred to 1/4 strength fresh Hoagland solutions, and their foliage was exposed to 50 mg L^-1^ Fe_3_O_4_ NPs (**a**) or FeSO_4_ (**b**) for 21 days. FeNP, 50 mg·L^-1^ Fe_3_O_4_ NPs; Fe, 50 mg·L^-1^ FeSO_4_. Up, upregulated metabolites; down, downregulated metabolites.

**
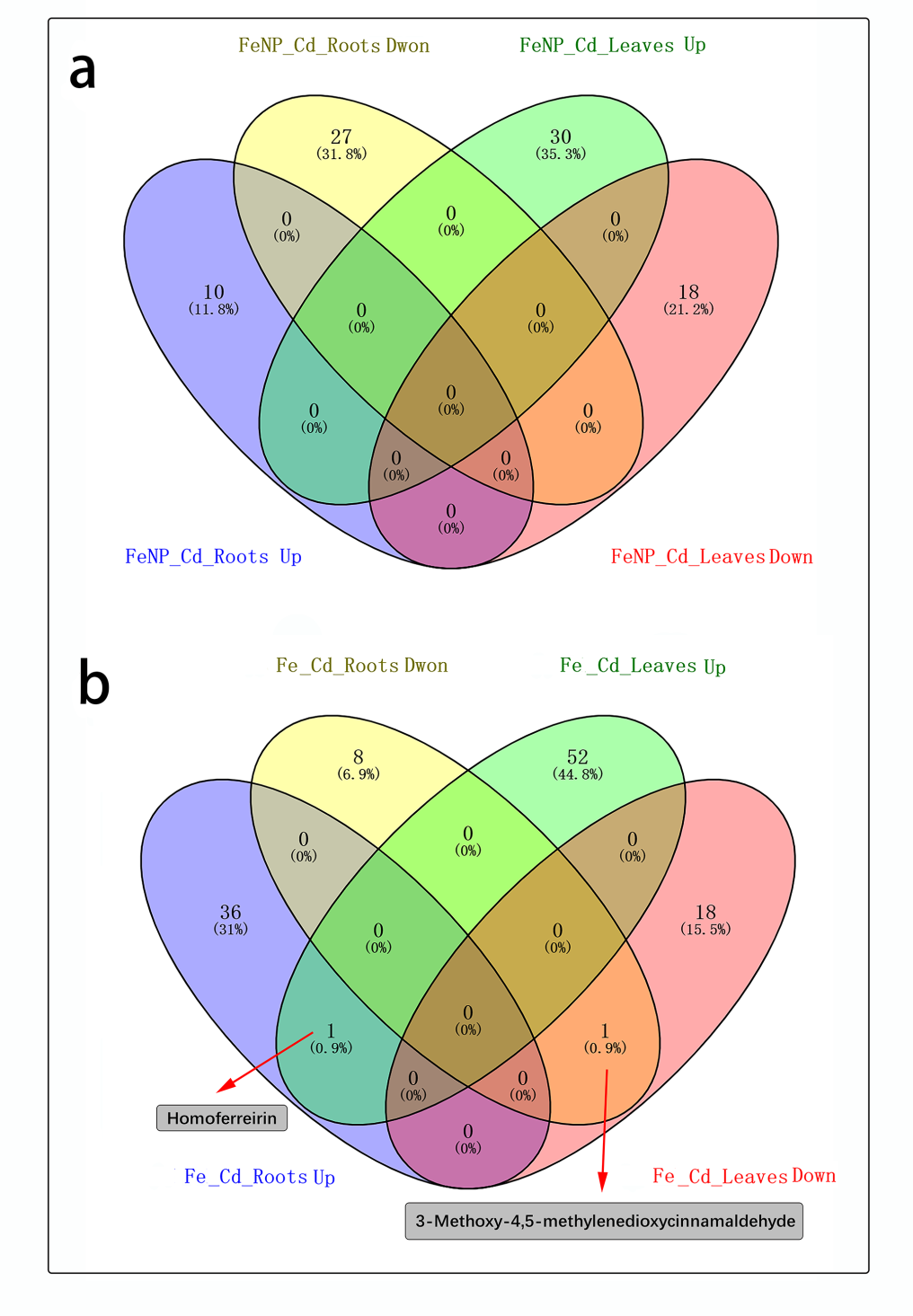
Figure S8. Venn analysis of differentially accumulated metabolites in the roots and leaves of Cd-treated tobacco seedlings exposed to Fe_3_O_4_ NPs or FeSO_4_.** Four-week-old tobacco seedlings were transferred to 1/4 strength fresh Hoagland solutions supplemented with 5 μM CdCl_2_, and their foliage was exposed to 50 mg L^-1^ Fe_3_O_4_ NPs (**a**) or FeSO_4_ (**b**) for 21 days. Cd, 5 μM Cd; FeNP_Cd, 50 mg·L^-1^ Fe_3_O_4_ NPs+Cd; Fe_Cd, 50 mg·L^-1^ FeSO_4_+Cd. Up, upregulated metabolites; down, downregulated metabolites.

**
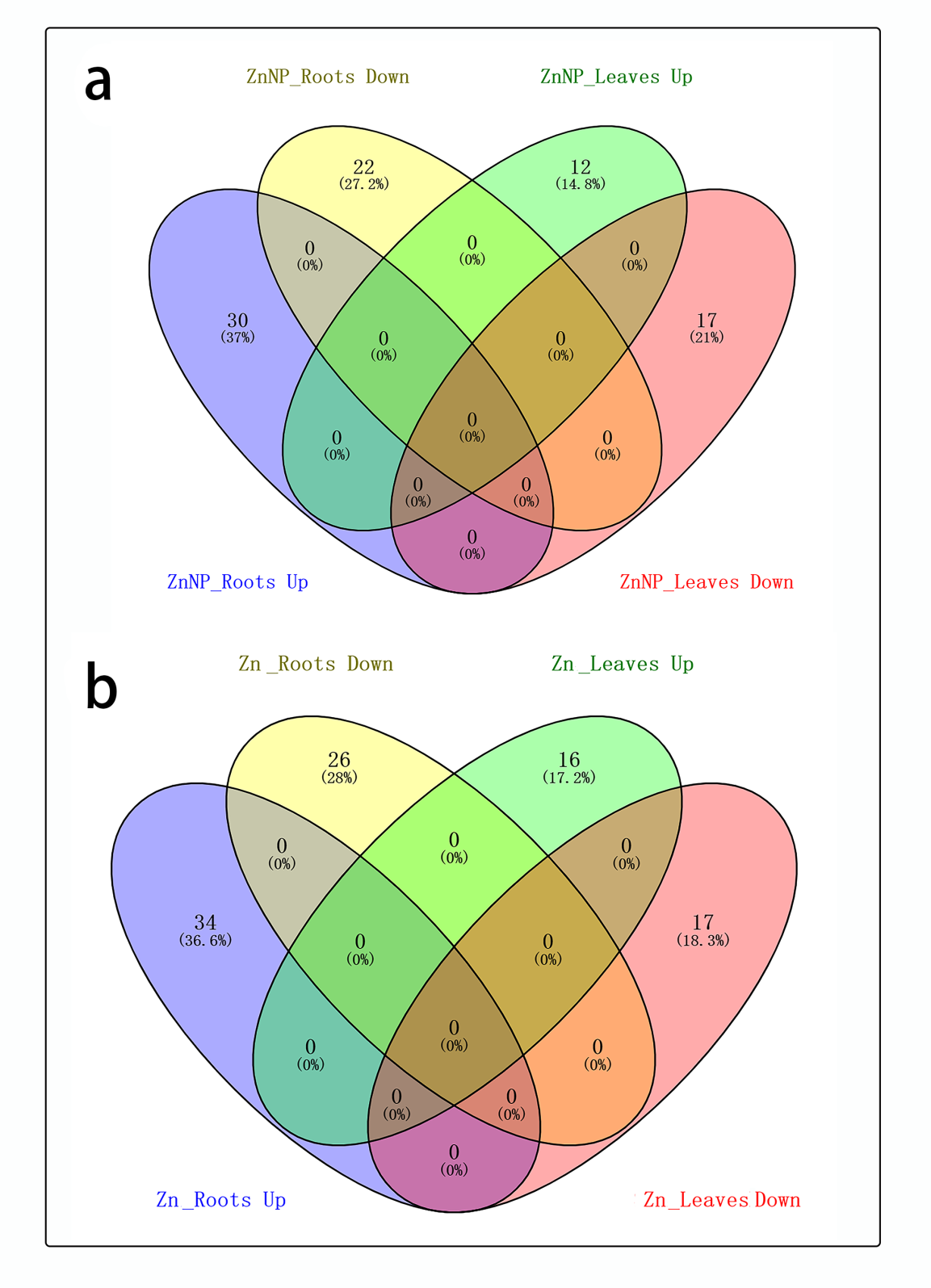
Figure S9. Venn analysis of differentially accumulated metabolites in the roots and leaves of tobacco seedlings exposed to ZnO NPs or ZnSO_4_.** Four-week-old seedlings were transferred to 1/4 strength fresh Hoagland solutions, and their foliage was exposed to 50 mg·L^-1^ ZnO NPs (**a**) or ZnSO_4_ (**b**) for 21 days. ZnNP, 50 mg·L^-1^ ZnO NPs; Zn, 50 mg·L^-1^ ZnSO_4_. Up, upregulated metabolites; down, downregulated metabolites.

**
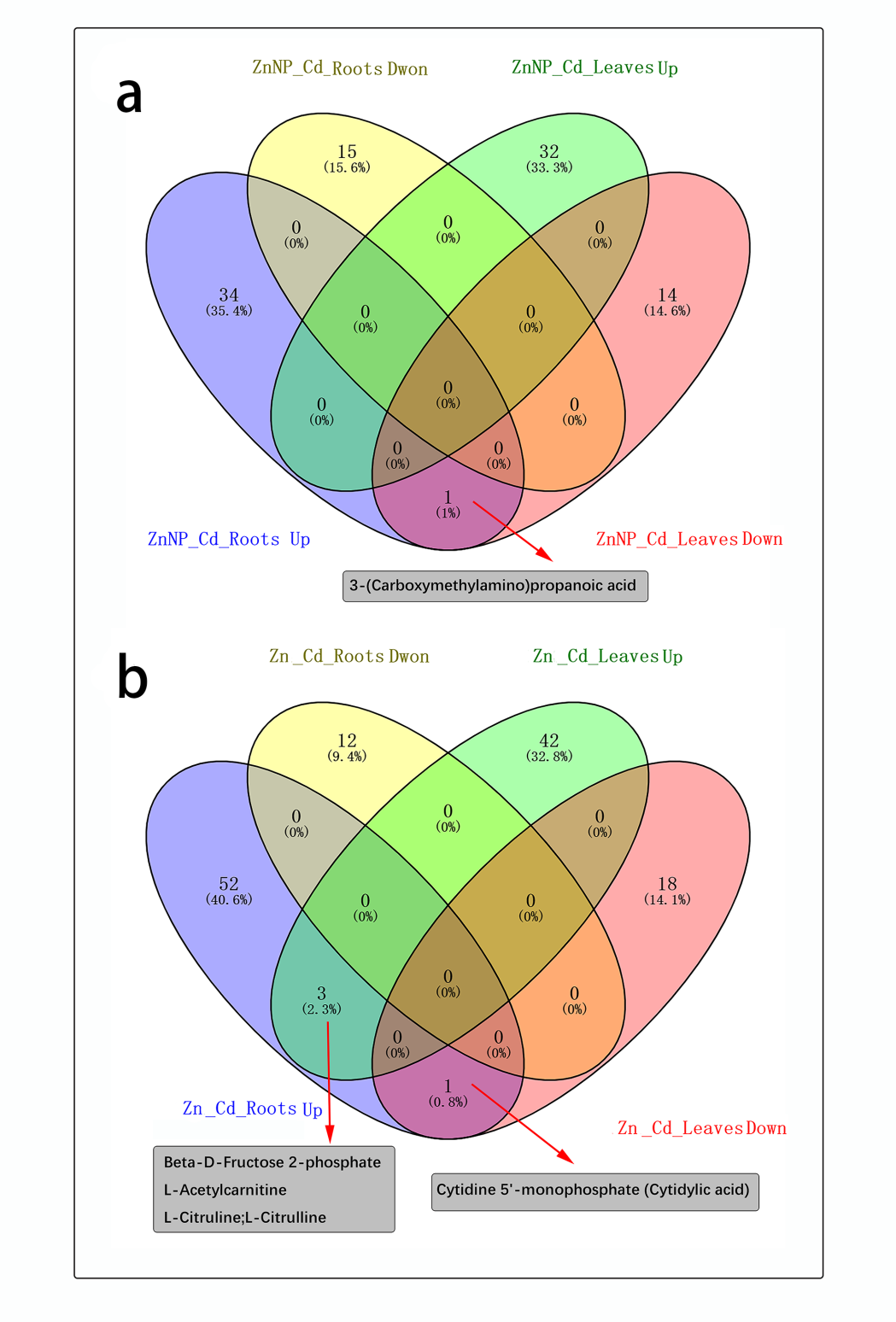
Figure S10. Venn analysis of differentially accumulated metabolites in the roots and leaves of Cd-treated tobacco seedlings exposed to ZnO NPs or ZnSO_4_.** Four-week-old tobacco seedlings were transferred to 1/4 strength fresh Hoagland solutions supplemented with 5 μM CdCl_2_, and foliar exposed to 50 mg·L^-1^ ZnO NPs (**a**) or ZnSO_4_ (**b**) for 21 days. Cd, 5 μM Cd; ZnNP_Cd, 50 mg·L^-1^ ZnO NPs+Cd; Zn_Cd, 50 mg·L^-1^ ZnSO_4_+Cd. Up, upregulated metabolites; down, downregulated metabolites.

**
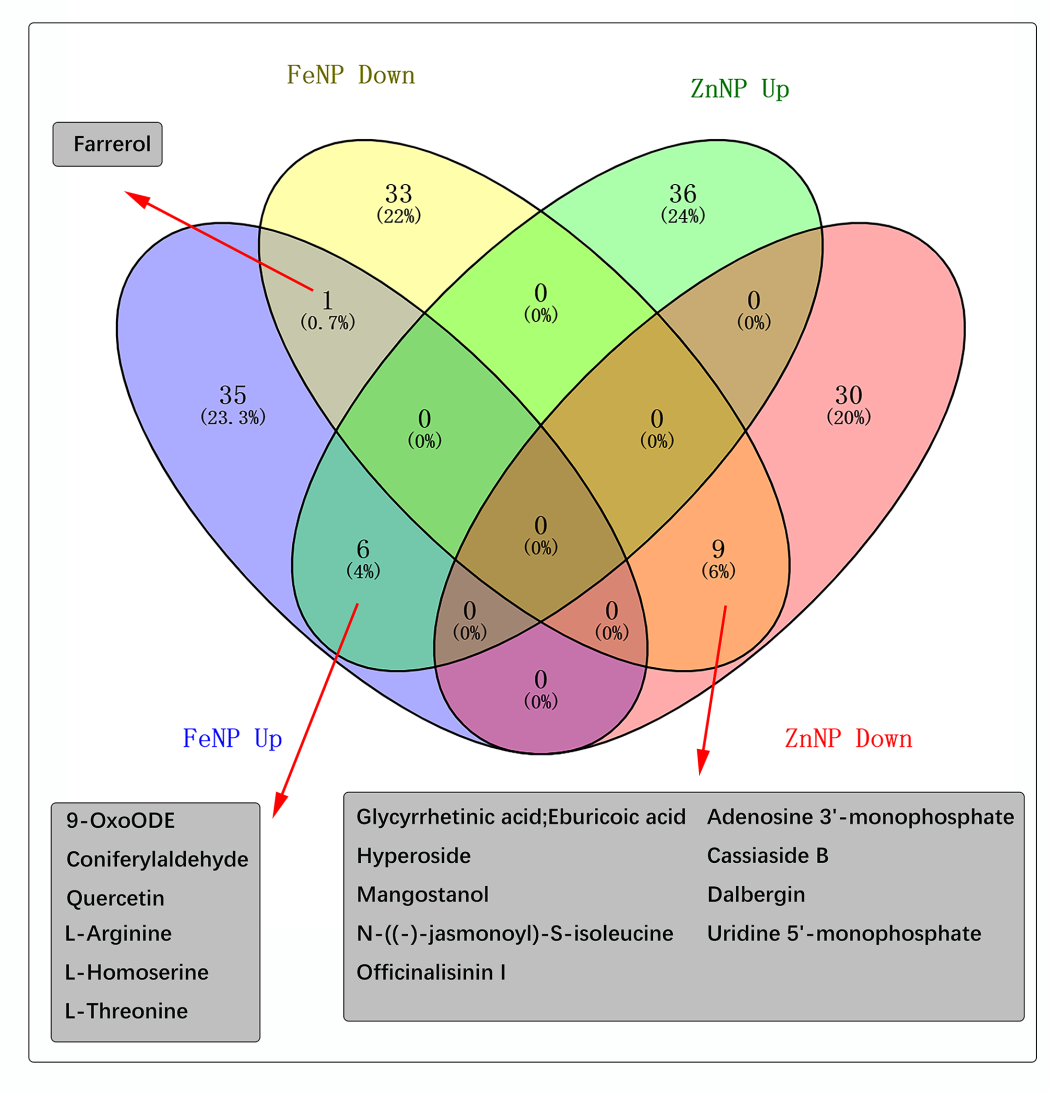
Figure S11. Venn analysis of differentially accumulated metabolites in the roots and/or leaves of tobacco seedlings exposed to Fe_3_O_4_ or ZnO NPs.** FeNP, 50 mg·L^-1^ Fe_3_O_4_ NPs; ZnNP, 50 mg·L^-1^ ZnO NPs. Up, upregulated metabolites; Down, downregulated metabolites.

**
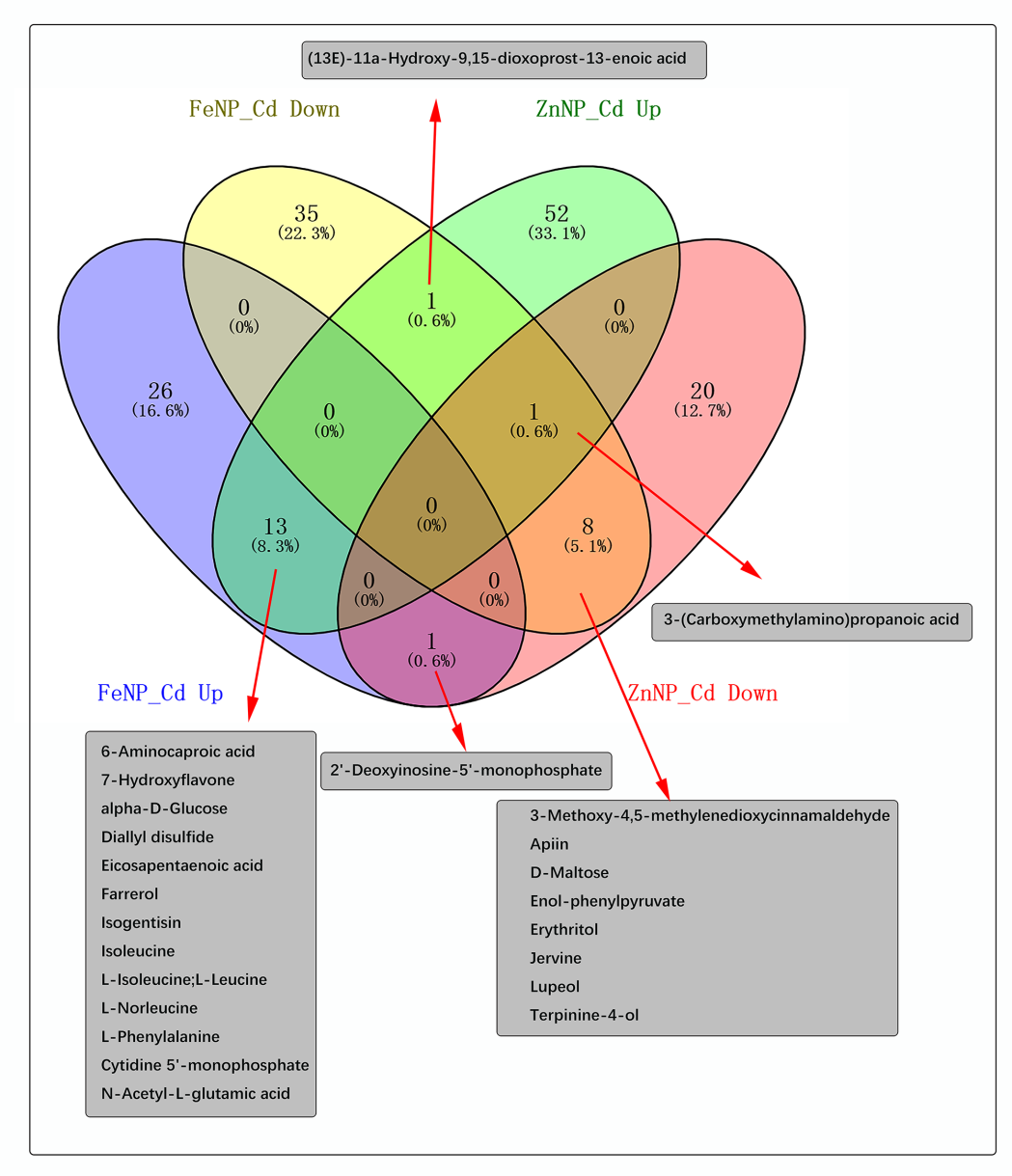
Figure S12. Venn analysis of differentially accumulated metabolites in the roots and/or leaves of Cd-treated tobacco seedlings exposed to Fe_3_O_4_ or ZnO NPs.** Cd, 5μM Cd; FeNP_Cd, 50 mg·L^-1^ Fe_3_O_4_ NPs+Cd; ZnNP_Cd, 50 mg·L^-1^ ZnO NPs+Cd. Up, upregulated metabolites; Down, downregulated metabolites.

**
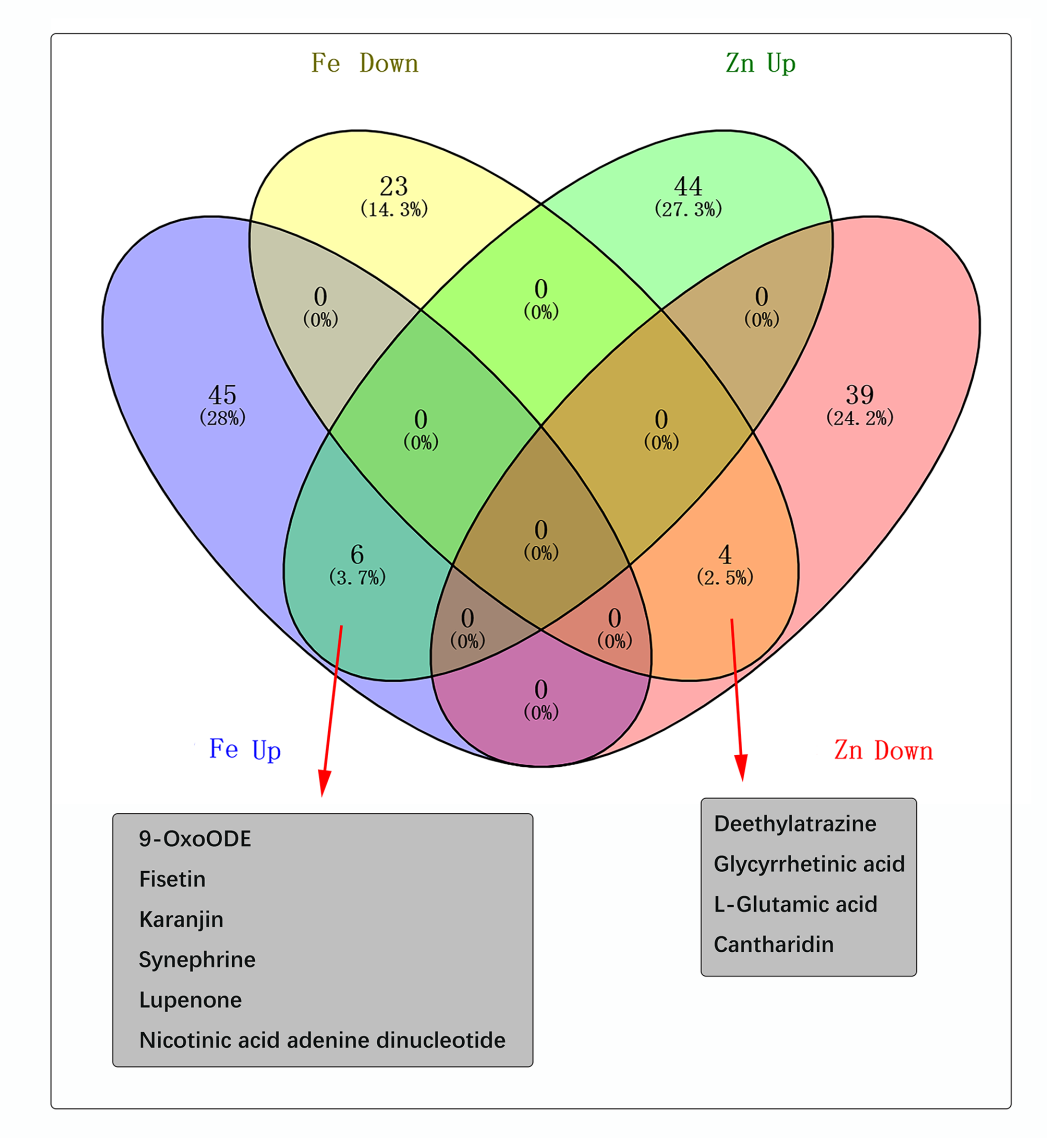
Figure S13. Venn analysis of differentially accumulated metabolites in the roots and/or leaves of tobacco seedlings exposed to FeSO_4_ or ZnSO_4_.** Fe, 50 mg·L^-1^ FeSO_4_; Zn, 50 mg·L^-1^ ZnSO_4_. Up, upregulated metabolites; Down, downregulated metabolites.

**
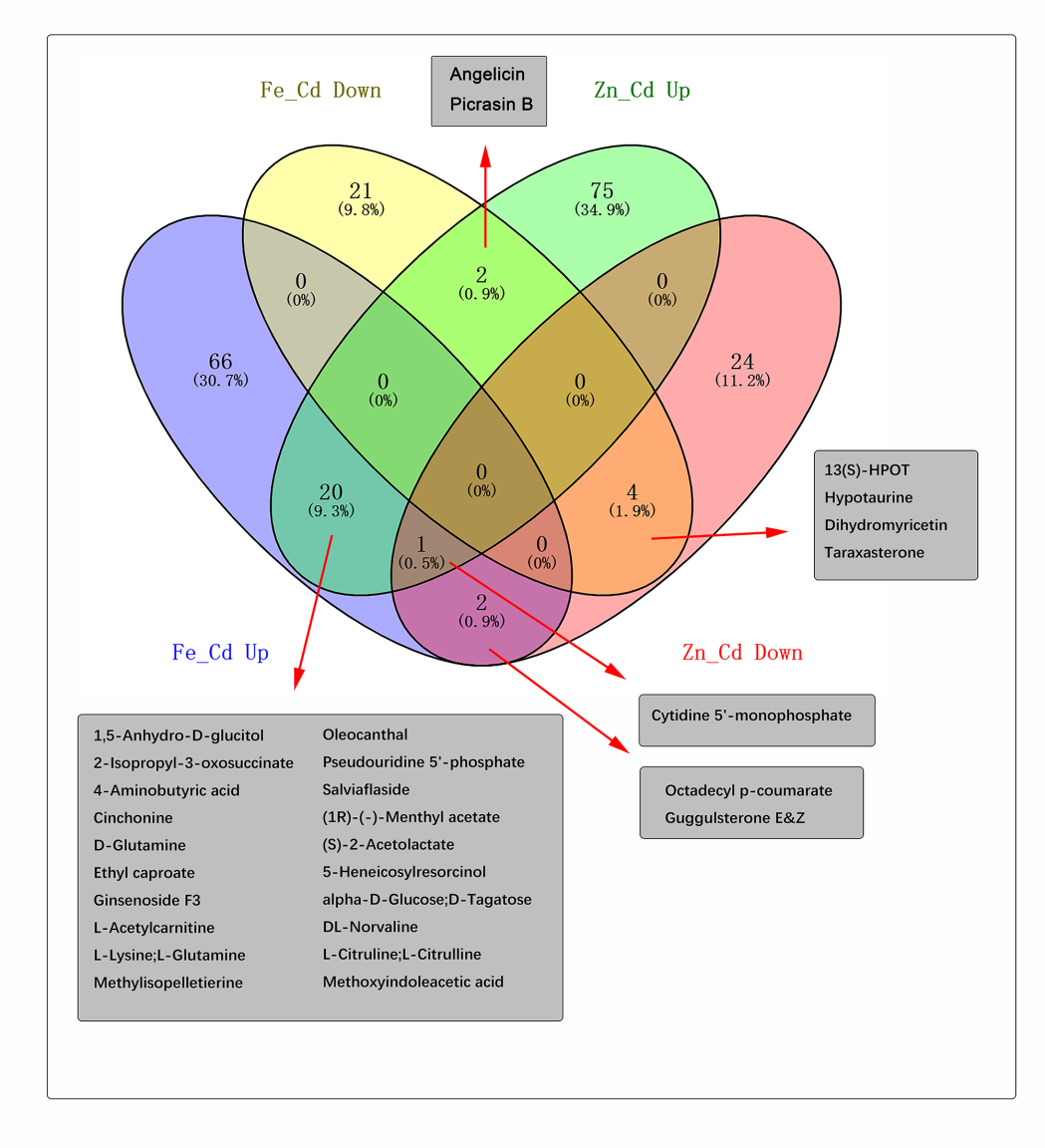
Figure S14. Venn analysis of differentially accumulated metabolites in the roots and/or leaves of Cd-treated tobacco seedlings exposed to FeSO_4_ or ZnSO_4_.** Cd, 5μM Cd; Fe_Cd, 50 mg·L^-1^ FeSO_4_+Cd; Zn_Cd, 50 mg·L^-1^ ZnSO_4_+Cd. Up, upregulated metabolites; Down, downregulated metabolites.

**
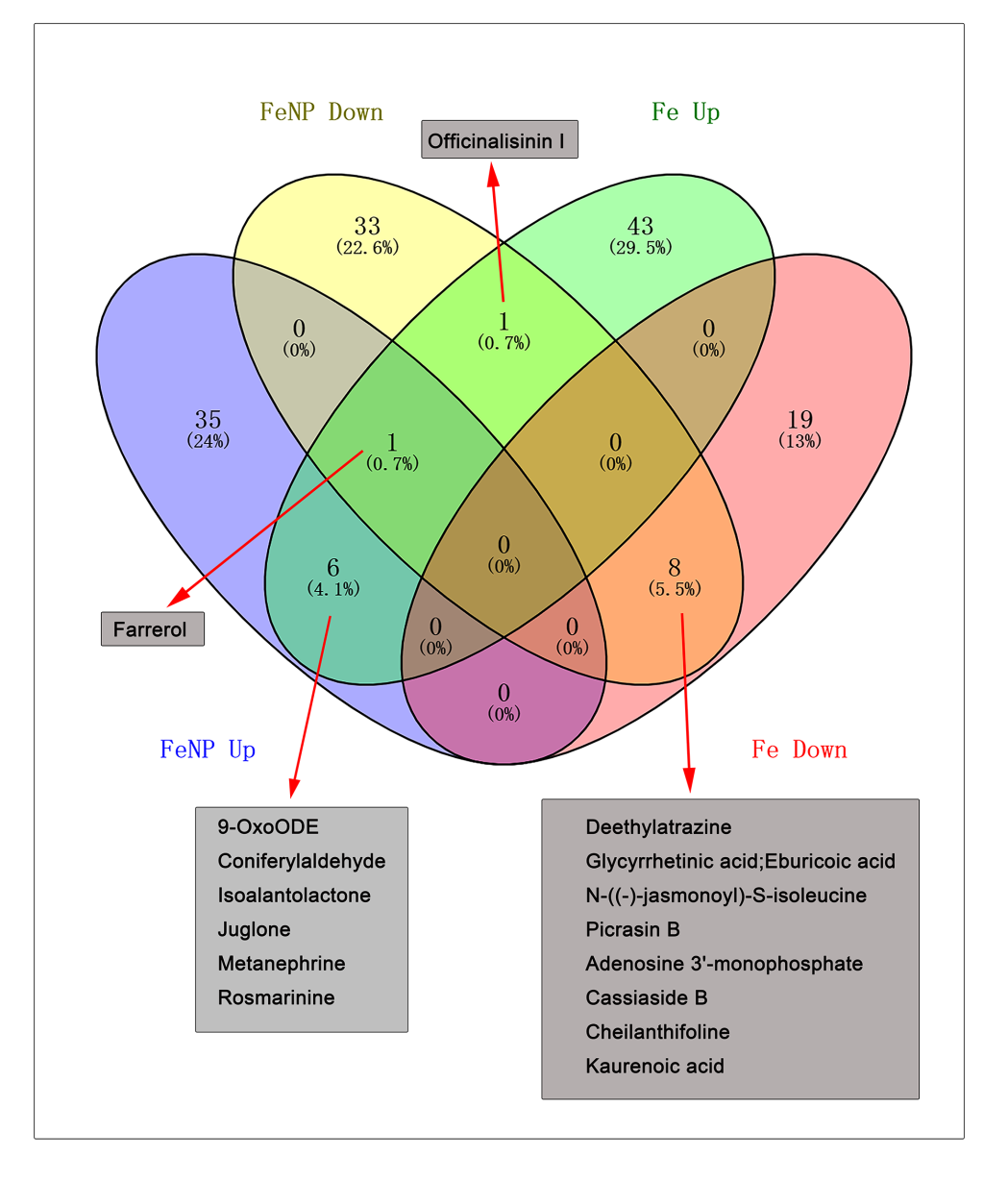
Figure S15. Venn analysis of differentially accumulated metabolites in the roots and/or leaves of tobacco seedlings exposed to Fe_3_O_4_ NPs or FeSO_4_.** FeNP, 50 mg·L^-1^ Fe_3_O_4_ NPs; Fe, 50 mg·L^-1^ FeSO_4_. Up, upregulated metabolites; Down, downregulated metabolites.

**
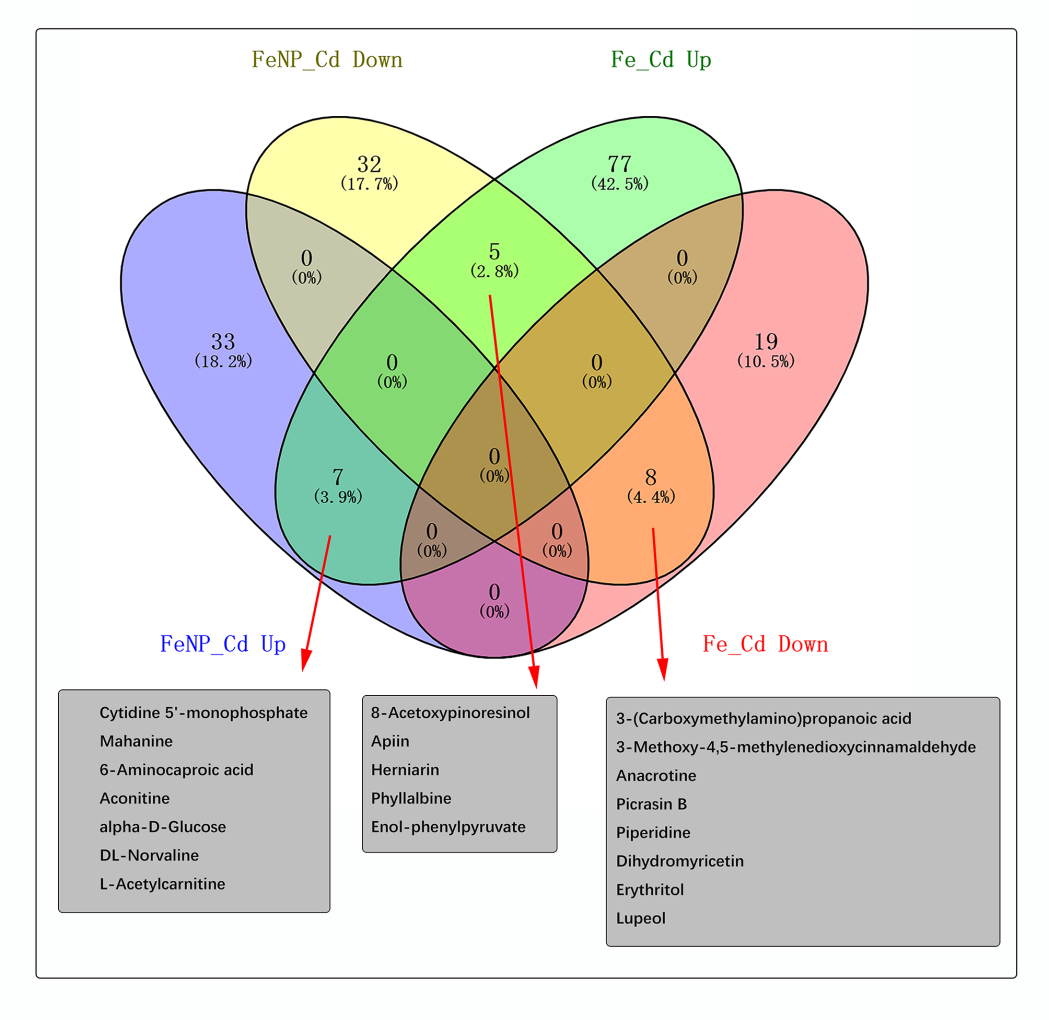
Figure S16. Venn analysis of differentially accumulated metabolites in the roots and/or leaves of Cd-treated tobacco seedlings exposed to Fe_3_O_4_ NPs or FeSO_4_.** Cd, 5μM Cd; FeNP_Cd, 50 mg·L^-1^ Fe_3_O_4_ NPs+Cd; Fe_Cd, 50 mg·L^-1^ FeSO_4_+Cd. Up, upregulated metabolites; Down, downregulated metabolites.

**
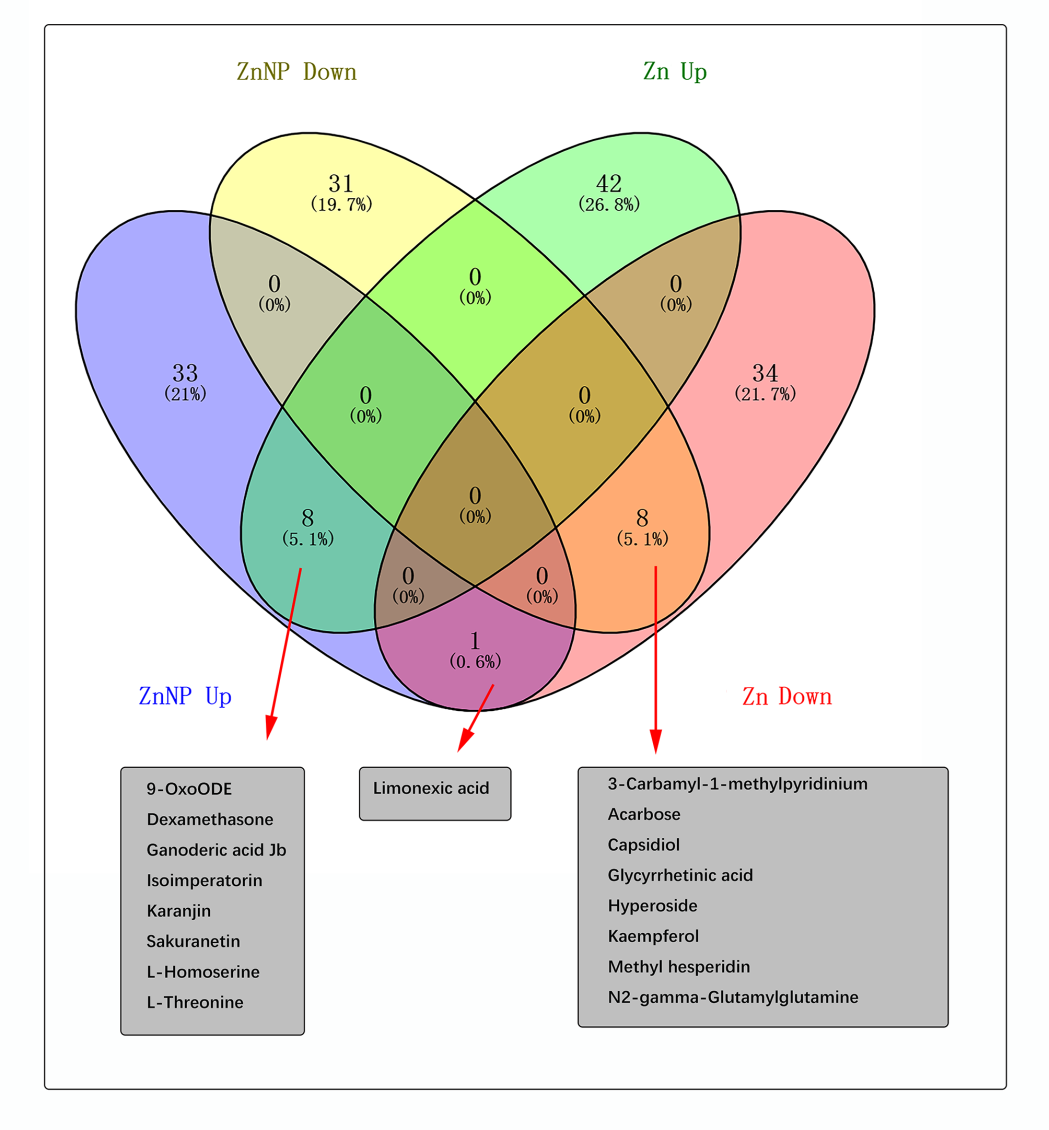
Figure S17. Venn analysis of differentially accumulated metabolites in the roots and/or leaves of tobacco seedlings exposed to ZnO NPs or ZnSO_4_.** ZnNP, 50 mg·L^-1^ ZnO NPs; Zn, 50 mg·L^-1^ ZnSO_4_. Up, upregulated metabolites; Down, downregulated metabolites.

**
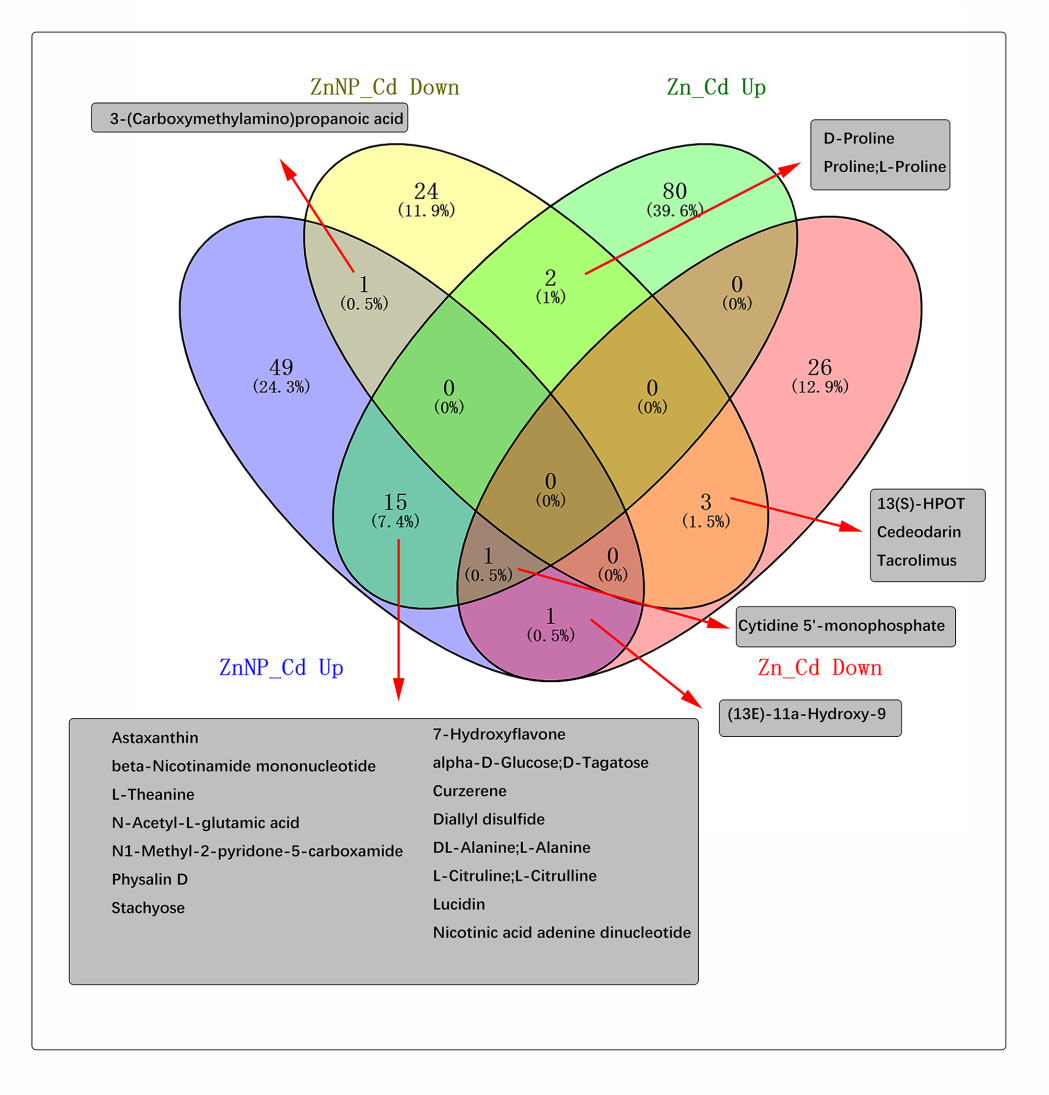
Figure S18. Venn analysis of differentially accumulated metabolites in the roots and/or leaves of Cd-treated tobacco seedlings exposed to ZnO NPs or ZnSO_4_.** Cd, 5μM Cd; ZnNP_Cd, 50 mg·L^-1^ ZnO NPs+Cd; Zn_Cd, 50 mg·L^-1^ ZnSO_4_+Cd. Up, upregulated metabolites; downregulated metabolites.
