# Supplementary material for: Comparative physiological and metabolomic analyses reveal that Fe3O4 and ZnO nanoparticles alleviate Cd toxicity in tobacco
Source: J Nanobiotechnology. 2022 Jun 27;20:302. doi: 10.1186/s12951-022-01509-3 (PMC9235244; doi:10.1186/s12951-022-01509-3)
Supplement: Supplementary file 9 — Additional file 9: Text S1. Supporting information. [file 12951_2022_1509_MOESM9_ESM.docx]

**Supporting Information Text S1**

**Metabolomics profiling using UHPLC-MS/MS**

**Metabolite extraction**

Samples exposed to 50 mg·L^-1^ Fe_3_O_4_ NPs, FeSO_4,_ ZnO NPs, or ZnSO_4_ with or without 5 μM CdCl_2_ were harvested for the indicated time and quickly frozen in liquid nitrogen and then stored at -80°C before use. First, the freeze-dried samples were crushed using a mixer mill for 60 s at 60 Hz. Then, 100 mg of powder from each sample was extracted using an Eppendorf tube containing 3 ml of 70% CH_3_OH (methanol/water, v:v=3:1). Next, the samples were homogenized for 30 s; two tiny steel balls were added for grinding at 35 Hz for 4 min, followed by ultrasonication in an ice bath for 15 min, and overnight shaking at 4°C. Subsequently, the samples were centrifuged at 12000 rpm for 15 min at 4°C, and 1.8 ml of supernatant was collected following nitrogen blow-drying. Next, dried samples were Resuspended in 900 μL of 50% CH_3_OH for 15 min in an ice bath and centrifuged at 12000 rpm for 15 min at 4°C. Lastly, the supernatants were filtered using a 0.22 μm filter membrane, and the resulting supernatants were diluted 10 times with 70% CH_3_OH (methanol/water, v:v=3:1), vortexed for 30 s and transferred to 2 ml glass vials. Equal aliquots of the supernatants from all samples were mixed for quality control (QC) samples. All the samples were stored at -80 °C until the analysis of ultra-performance liquid chromatography-tandem mass spectrometry (UPLC-MS/MS).

**UHPLC- MS analysis**

UHPLC-MS/MS analysis was conducted using a UHPLC system with a Phenomenex Kinetex column coupled to a Triple TOF 6600 instrument (QTOF, AB Sciex) belonging to Biotree Biomedical Technology Co., Ltd. Shanghai, China. First, sample separation was conducted using a Waters ACQUITY UPLC HSS T3 column (100 × 2.1 mm, 1.8 *μ*m). Then, aqueous 0.1% formic acid (v:v) and acetonitrile were used for mobile phases A and B, respectively. Sample measurement was conducted with a gradient program. The column and the autosampler temperatures were set at 40°C and 4°C, respectively. The injection volume was 2 μL, and MS/MS spectra were acquired on an information-dependent basis (IDA) during an LC/MS experiment. An AB Sciex QTrap 6500 mass spectrometer was applied for assay development based on set ESI parameters (ion spray voltage: +5000/-4500 V, curtain gas: 35 psi, temperature: 400°C, ion source gas 1:60 psi, ion source gas 2: 60 psi, and DP: ±100 V).

**Data preprocessing and annotation**

MRM data acquisition and processing were employed using SCIEX Analyst Work Station Software (v1.6.3). First, the raw MS data were converted to the TXT format using MSconverter. Then a data matrix consisting of the retention time (RT), mass-to-charge ratio (m/z) values, and peak intensity was generated, followed by metabolite identification with an in-house R program and database. Finally, the mass spectra were compared to known and commercially available mass spectral libraries, and the relative metabolite content was determined by checking the peak area (mm^2^).

After the data were log_2_-transformed and mean-centering processed, orthogonal projections to latent structures-discriminant analysis (OPLS-DA, MetaboAnalystR) were performed to present group differences. Hierarchical cluster analysis (HCA) was conducted for different samples and metabolites, shown as heat maps and dendrograms. The threshold was set to variable importance in projection (VIP) ≧ 1 and a *P-value* < 0.05 to determine the significantly changed metabolites between groups. For correlation analysis of DAMs and growth parameters among different groups, the DAM mean values of each treatment and growth parameters were calculated, and Pearson correlation coefficients were calculated using R 3.5.1 software according to a Student’s *t*-test. The DAMs were significantly correlated with the growth parameter at *r*>0.75 or *r<* -0.75 and *P*<0.001.
